# Supplementary material for: ACE: A Versatile Contrastive Learning Framework for Single-cell Mosaic Integration
Source: Genomics Proteomics Bioinformatics. 2025 Aug 4;23(4):qzaf062. doi: 10.1093/gpbjnl/qzaf062 (PMC12582371; doi:10.1093/gpbjnl/qzaf062)
Supplement: qzaf062_Supplementary_Data [file qzaf062_supplementary_data.zip › supplementary material captions.docx]

**Supplementary material**

**File S1 Supplementary text for ACE**

**Figure S1** **Examples of constructing positive and negative pairs for contrastive learning**

**A.** Construction examples of ACE-align in two-modality and three-modality cases, respectively. **B.** Construction example of ACE-spec.

**Figure S2 Illustration of ACE-spec’s workflow**

**A.** Input and output of ACE-spec. **B**. The embedding imputation process of ACE-spec. ACE-spec imputes the missing modality-specific embeddings in each batch and then averages the embeddings from all modalities to get the final embedding for each batch.

**Figure S3 Bar plots of bi-modal benchmarking results**

**A**–**D.** show the scores on the BM-CITE (**A**), PBMC-Mult (**B**), CITE (**C**), and Multiome (**D**) datasets, respectively.

**Figure S4 UMAP plots of embeddings in bi-modal case 1 from all compared methods**

In each panel, cells in the first row are colored by cell types and colored by modal labels (batch labels) in the second row. **A.** UMAP plots on the BM-CITE datasets. **B.** UMAP plots on the PBMC-Mult dataset.

**Figure S5 Mosaic integration benchmark on Multiome dataset of bi-modal case 2**

We labeled the top three methods for each score.

**Figure S6 UMAP plots of embeddings in bi-modal case 2 from all compared methods**

In each panel, cells in the first row are colored by cell types, colored by batch labels in the second row, and colored by modal labels in the third row. **A.** UMAP plots on the CITE dataset. **B.** UMAP plots on the Multiome dataset.

**Figure S7 Specific metrics and scores in bi-modal mosaic integration benchmark**

1. Bio-conservation scores, batch correction scores, and overall scores in case 3 and case 4. **B.** Specific metrics in case 3 and case 4. They are not min-max scaled. **C.** Specific metrics in experiment of removing cells from bridge batches. They are not min-max scaled.

**Figure S8 Bar plots of tri-modal benchmarking results**

**A and B.** The scores on the DOGMA and CITE-ASAP datasets, respectively.

**Figure S9 UMAP plots of embeddings in tri-modal integration tasks from all compared methods**

**A.** UMAP plots on the DOGMA dataset. Cells in the first row are colored by cell types, colored by modal (batch) labels in the second row. **B.** UMAP plots on the Multiome dataset. Cells in the first row are colored by cell types, colored by batch labels in the second row, and colored by modal labels in the third row.

**Figure S10 Specific metrics and scores in tri-modal mosaic integration benchmark**

**A.** Bio-conservation scores, batch correction scores, and overall scores in tri-modal case 4. **B.** Specific metrics in tri-modal case 4. They are not min-max scaled. **C.** All scores in experiment of removing cells from bridge batches. **D.** Specific metrics in experiment of removing cells from bridge batches. They are not min-max scaled.

**Figure S11 Analysis of ACE-spec’s results on the CITE-ASAP dataset**

**A.** UMAP plots based on raw modality profiles. Cells are colored by clustering labels from ACE-spec. **B.** UMAP plots of WNN results on the batches measured with CITE-seq and ASAP-seq. Cells are colored by clustering labels from ACE-spec. **C.** UMAP plots of ACE-spec’s embedding on the batches measured with ASAP-seq. In the left, cells are colored by the clustering labels; heatmap displaying the expression level of surface protein TCR Vα7.2 in these cells. **D.** Activity heatmap of known marker motifs. NK: EOMES, TBX21; B: SPI1, EBF1, IRF8; Naïve CD4 T: CTCF, TCF7L2; CD4+ Memory T: GATA3; T regulatory (Treg): JUNB, FOSL2; Naïve CD8 T: CTCF, TCF7L2, LEF1; CD8+ memory T: TBX21, EOMES; MAIT: RORA, RORB, RORC; gdT: RORC, RORA, RORC, TBX21, EOMES; CD14 Monocytes: CEBPB, CEBPD, CEBPE, CEBPG; cDC: SP1, JUNB; pDC: RUNX2, SPI1, SPIB; HSPC: GATA1::TAL1, GATA2, SPI1.

**Figure S12 KEGG pathway enrichment analysis on the top 200 differentially expressed genes for each ACE-spec identified cluster**

We labelled each cluster with our annotations.

**Figure S13 UMAP plots of raw CITE2, VP-RNA, CYTOF datasets and embeddings from ACE-spec**

In each panel, cells are colored by cell type annotations in the first row and colored by batch labels in the second row. **A.** UMAP visualizations of raw RNA, protein data of CITE2 dataset, WNN results, and embeddings from ACE-spec. **B.** UMAP visualizations of raw VP-RNA dataset and embeddings from ACE-spec. **C.** UMAP visualizations of raw CYTOF dataset and embeddings from ACE-spec.

**Figure S14 Expression heatmap of known markers genes in VP-RNA dataset**

**Figure S15 Parameter sensitivity experiment of ACE and comparison of time consumption among all methods**

**A.** ACE-align and ACE-spec’s sensitivity to parameters $d$ and $\tau$ with respect to iLISI, NMI, ARI, $\mathrm{FOSCTT}M_{0}$ $\mathrm{FOSCTT}M_{0}$, and matching scores in bi-modal case 2. **B.** ACE-align and ACE-spec’s sensitivity to parameters $d$ and $\tau$ with respect to iLISI, NMI, ARI in bi-modal case 4. **C.** ACE-spec’s sensitivity to parameter *k*. **D.** Comparison of time consumption (seconds) of all methods on multiple subsampled CITE2 dataset. Note that the time consumed by data loading and preprocessing/post-processing steps such as normalization, dimension reduction, and batch correction is not recorded.

**Figure S16 Analysis of modality gap phenomena**

**A.** For each cell, Manhattan distances were calculated to the nearest neighbor within the same modality (intra-modality) and to its paired embedding in the other modality (inter-modality), using embeddings derived from InfoNCE and our proposed loss functions. **B.** For each cell, Euclidean distances were calculated to the nearest neighbor within the same modality (intra-modality) and to its paired embedding in the other modality (inter-modality), using embeddings derived from InfoNCE and our proposed loss functions.

**Figure S17 UMAP visualizations of embedding on BM-CITE and CITE datasets, derived from our proposed loss function and InfoNCE with different temperature value settings**

For BM-CITE dataset, cells are colored by cell types in the first row and colored by modal (batch) labels in the second row. For CITE dataset, cells are colored by cell types in the first row, colored by modal labels in the second row, and colored by batch labels in the third row.

**Figure S18 Analysis of modality gap phenomena within a larger area**

**A.** For each cell, Manhattan distances to the top 10 nearest neighbors within intra-modality and inter-modality were calculated based on embeddings derived from InfoNCE and our loss function. **B.** For each cell, Euclidean distances to the top 10 nearest neighbors within intra-modality and inter-modality were calculated based on embeddings derived from InfoNCE and our loss function.

**Figure S19 Varying UMAP hyperparameters, number of nearest neighbors, and minimum distance, in visualizing ACE-align, ACE-spec, Cobolt, and scMoMaT’s embeddings on BM-CITE dataset**

Cells are colored by batch labels.

**Figure S20 Varying UMAP hyperparameters, number of nearest neighbors, and minimum distance, in visualizing ACE-align, ACE-spec, Cobolt, and scMoMaT’s embeddings on BM-CITE dataset**

Cells are colored by cell type labels.

**Figure S21 Varying UMAP hyperparameters, number of nearest neighbors, and minimum distance, in visualizing ACE-align, ACE-spec, Cobolt, and scMoMaT’s embeddings on PBMC-Mult dataset**

Cells are colored by batch labels.

**Figure S22 Varying UMAP hyperparameters, number of nearest neighbors, and minimum distance, in visualizing ACE-align, ACE-spec, Cobolt, and scMoMaT’s embeddings on PBMC-Mult dataset**

Cells are colored by cell type labels.

**Figure S23 Varying UMAP hyperparameters, number of nearest neighbors, and minimum distance, in visualizing ACE-spec’s embeddings on CITE-ASAP dataset**

**A and B.** Cells are colored by cell type and cluster labels in (**A**) and (**B**), respectively.

**Figure S24 Varying UMAP hyperparameters, number of nearest neighbors, and minimum distance, in visualizing ACE-spec’s embeddings on the VP dataset**

**A and B.** Cells are colored by cell type and cluster labels in (**A**) and (**B**), respectively.

**Figure S25 Ablation study on our proposed contrastive learning loss**

**A.** Comparison between our loss function and InfoNCE on BM-CITE dataset for different temperature value settings. **B.** Comparison between our loss function and InfoNCE on CITE dataset for different temperature value settings. **C.** Comparison between our loss function and InfoNCE on CITE dataset for different input numbers of protein features.

**Figure S26 Evaluation of the impact of four batch correction methods on the performance of ACE-align and ACE-spec in CITE-5%/10% and Multiome-5%/10% datasets**

The terms “-5%” and “-10%” denote subsampled datasets with sampling ratios of 5% and 10%, respectively.

**Figure S27 UMAP visualizations of batch corrected modality inputs processed from four batch correction methods**

The terms “-5%” and “-10%” denote subsampled datasets with sampling ratios of 5% and 10%, respectively.

**Figure S28 Evaluation of the impact of four batch correction methods on the performance of ACE-align and ACE-spec in DOGMA dataset**

**A.** Values of specific evaluation metrics, including NMI, ARI, modal iLISI, batch iLISI, FOSCTTM, and matching score. **B.** Bio-conservation score, batch correction score, modality alignment score, and overall score.

**Figure S29 UMAP visualizations of batch corrected modality inputs processed from four batch correction methods**

**Figure S30 Evaluation of the impact of three batch correction methods on the performance of ACE-align and ACE-spec in CITE dataset**

**A.** Values of specific evaluation metrics, including NMI, ARI, modal iLISI, batch iLISI, FOSCTTM, and matching score. **B.** Bio-conservation score, batch correction score, modality alignment score, and overall score.

**Figure S31 Evaluation of the impact of three batch correction methods on the performance of ACE-align and ACE-spec in Muliome dataset**

**A.** Values of specific evaluation metrics, including NMI, ARI, modal iLISI, batch iLISI, FOSCTTM, and matching score. **B.** Bio-conservation score, batch correction score, modality alignment score, and overall score.

**Figure S32 Evaluation of the impact of three clustering algorithms on supervised metrics (NMI and ARI) for various integration methods across four datasets**

**Figure S33 Comparison of various integration methods combined with their optimal clustering algorithm, selected based on the highest NMI scores**

**Figure S34 Comparison of various integration methods using unsupervised metrics**

**A and B.** Silhouette score in (**A**) and Davies–Bouldin index in (**B**).

**Figure S35 UMAP visualizations of embeddings generated by various integration methods in CITE and Multiome datasets, with cells colored by annotated cell type and pseudotime**

**Figure S36 Evaluation of performance in imputing missing modality profiles**

**A.** Bar plots displaying overall scores of scVAEIT, MultiVI/totalVI, and ACE in different subtasks of different imputation scenarios. **B.** Box plots displaying metric scores of imputation for all individual features. **C.** UMAP plots for cells with imputed features (RNA and protein) on the BM-CITE dataset from ACE, scVAEIT, and totalVI. Cells are colored by cell types. **D.** UMAP plots for cells with imputed features and cells with corresponding ground truth of profiles. For each feature, cells are colored by cell types (the same as **C**) in the left and colored by their source in the right.

**Figure S37 UMAP plots of imputed features on PBMC-Multiome dataset**

**A.** UMAP plots for cells with imputed features (RNA and ATAC) on this dataset from ACE, scVAEIT, and MultiVI. Cells are colored by cell types. **B.** UMAP plots for cells with imputed features and cells with corresponding ground truth of profiles. Within each feature, cells are colored by cell types (the same as **A**) in the left and colored by their source in the right.

**Figure S38 UMAP plots of imputed features on the CITE dataset**

**A.** UMAP plots for cells with imputed features (RNA and protein) on this dataset from ACE, scVAEIT, and totalVI. Cells are colored by cell types. **B.** UMAP plots for cells with imputed features from different methods and cells with corresponding ground truth of profiles. Within each feature, cells are colored by cell types (the same as **A**) in the left and colored by their source in the right.

**Figure S39 UMAP plots of imputed features on the Multiome dataset**

**A.** UMAP plots for cells with imputed features (RNA and ATAC) on this dataset from ACE, scVAEIT, and MultiVI. Cells are colored by cell types. **B.** UMAP plots for cells with imputed features from different methods and cells with corresponding ground truth of profiles. Within each feature, cells are colored by cell types (the same as **A**) in the left and colored by their source in the right.
