# Supplementary material for: ACE: A Versatile Contrastive Learning Framework for Single-cell Mosaic Integration
Source: Genomics Proteomics Bioinformatics. 2025 Aug 4;23(4):qzaf062. doi: 10.1093/gpbjnl/qzaf062 (PMC12582371; doi:10.1093/gpbjnl/qzaf062)
Supplement: qzaf062_Supplementary_Data [file qzaf062_supplementary_data.zip › Figure S1.pptx]

## Slide 1
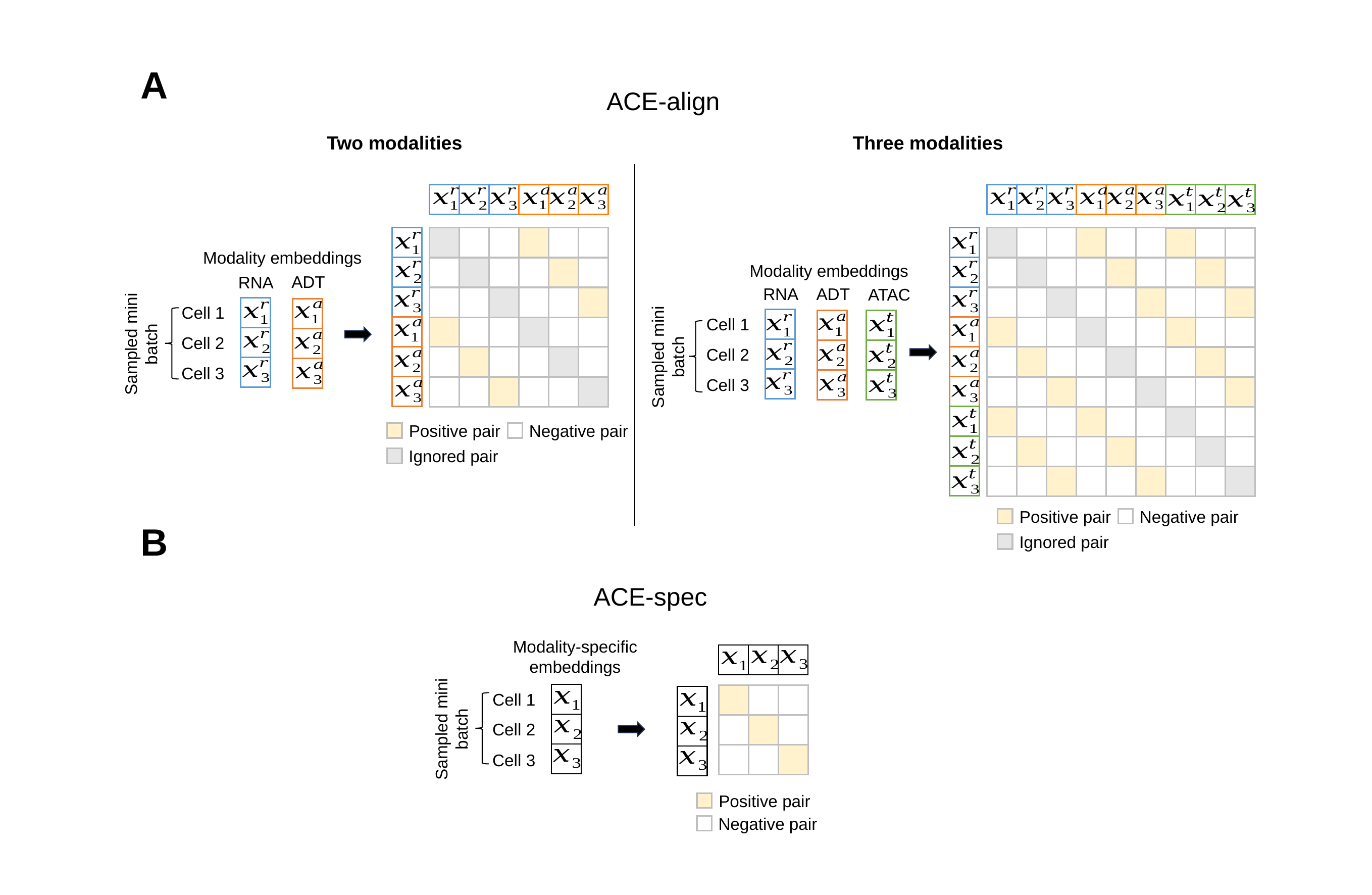

A
ACE-align
Two modalities
Three modalities
Positive pair
Negative pair
Ignored pair
Modality embeddings
Modality embeddings
ADT
RNA
ADT
RNA
ATAC
Cell 1
Cell 1
Sampled mini batch
Cell 2
Sampled mini batch
Cell 2
Cell 3
Cell 3
Positive pair
Negative pair
Ignored pair
B
ACE-spec
Modality-specific
embeddings
Cell 1
Sampled mini batch
Cell 2
Cell 3
Positive pair
Negative pair
