# Supplementary material for: ACE: A Versatile Contrastive Learning Framework for Single-cell Mosaic Integration
Source: Genomics Proteomics Bioinformatics. 2025 Aug 4;23(4):qzaf062. doi: 10.1093/gpbjnl/qzaf062 (PMC12582371; doi:10.1093/gpbjnl/qzaf062)
Supplement: qzaf062_Supplementary_Data [file qzaf062_supplementary_data.zip › Figure S2.pptx]

## Slide 1
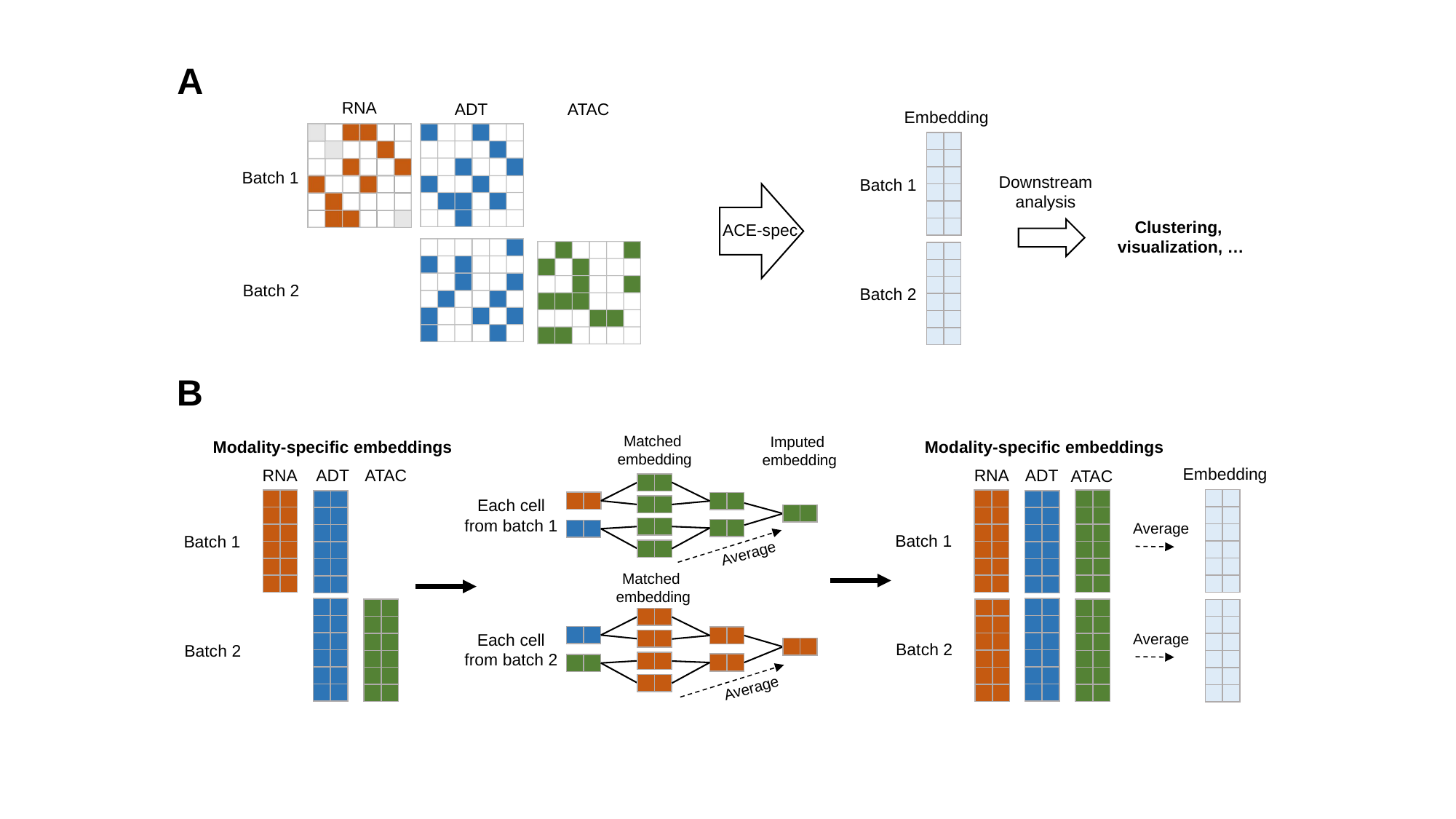

A
RNA
ADT
ATAC
Batch 1
Downstream analysis
Batch 1
ACE-spec
Clustering,
visualization, …
Batch 2
Batch 2
Embedding
B
Matched
embedding
Imputed
embedding
Modality-specific embeddings
Modality-specific embeddings
Embedding
RNA
ADT
ATAC
RNA
ADT
ATAC
Each cell from batch 1
Average
Batch 1
Batch 1
Average
Matched
embedding
Average
Each cell from batch 2
Batch 2
Batch 2
Average
