# Supplementary material for: ACE: A Versatile Contrastive Learning Framework for Single-cell Mosaic Integration
Source: Genomics Proteomics Bioinformatics. 2025 Aug 4;23(4):qzaf062. doi: 10.1093/gpbjnl/qzaf062 (PMC12582371; doi:10.1093/gpbjnl/qzaf062)
Supplement: qzaf062_Supplementary_Data [file qzaf062_supplementary_data.zip › Figure S4.pptx]

## Slide 1
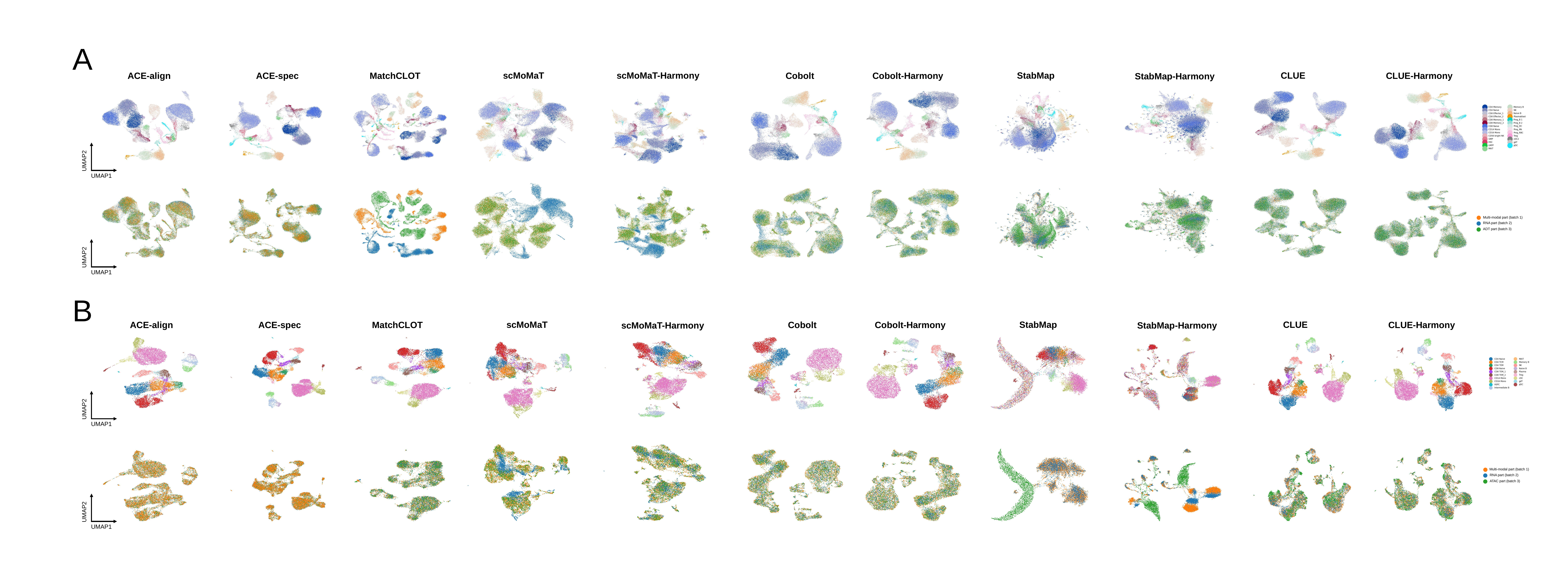

A
scMoMaT
scMoMaT-Harmony
StabMap
CLUE
ACE-align
ACE-spec
MatchCLOT
Cobolt
Cobolt-Harmony
CLUE-Harmony
StabMap-Harmony
UMAP2
UMAP1
Multi-modal part (batch 1)
RNA part (batch 2)
ADT part (batch 3)
UMAP2
UMAP1
B
scMoMaT
StabMap
CLUE
ACE-align
ACE-spec
MatchCLOT
Cobolt
Cobolt-Harmony
CLUE-Harmony
scMoMaT-Harmony
StabMap-Harmony
UMAP2
UMAP1
Multi-modal part (batch 1)
RNA part (batch 2)
ATAC part (batch 3)
UMAP2
UMAP1
