# Supplementary material for: ACE: A Versatile Contrastive Learning Framework for Single-cell Mosaic Integration
Source: Genomics Proteomics Bioinformatics. 2025 Aug 4;23(4):qzaf062. doi: 10.1093/gpbjnl/qzaf062 (PMC12582371; doi:10.1093/gpbjnl/qzaf062)
Supplement: qzaf062_Supplementary_Data [file qzaf062_supplementary_data.zip › Figure S5.pptx]

## Slide 1
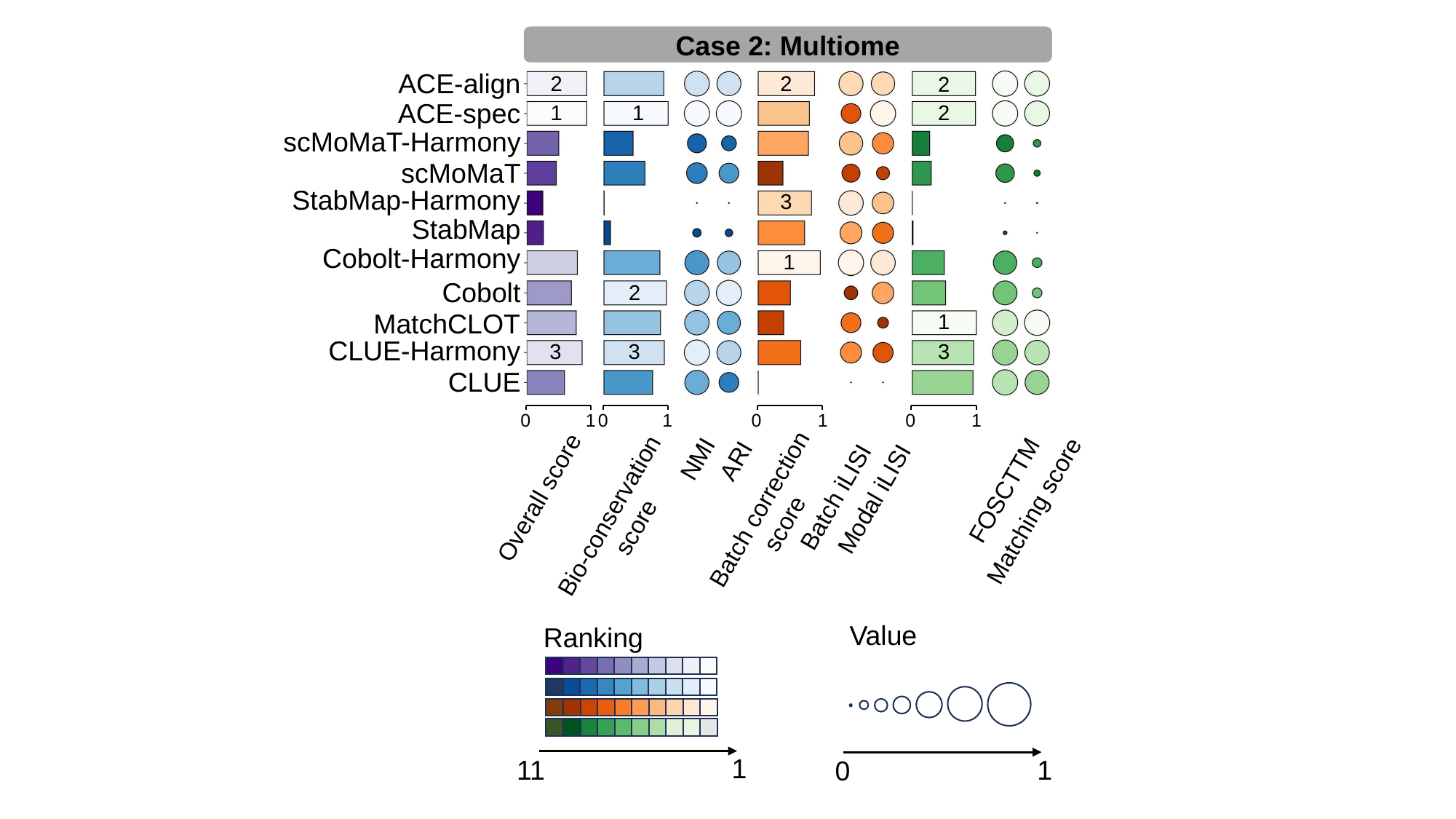

Case 2: Multiome
ACE-align
ACE-spec
scMoMaT-Harmony
scMoMaT
StabMap-Harmony
StabMap
Cobolt-Harmony
Cobolt
MatchCLOT
CLUE-Harmony
CLUE
0
1
0
1
0
1
0
1
ARI
NMI
Batch correction score
Bio-conservation
score
Batch iLISI
FOSCTTM
Modal iLISI
Overall score
Matching score
Value
Ranking
1
11
1
0
2
2
2
2
1
1
3
1
2
1
3
3
3
