# Supplementary material for: ACE: A Versatile Contrastive Learning Framework for Single-cell Mosaic Integration
Source: Genomics Proteomics Bioinformatics. 2025 Aug 4;23(4):qzaf062. doi: 10.1093/gpbjnl/qzaf062 (PMC12582371; doi:10.1093/gpbjnl/qzaf062)
Supplement: qzaf062_Supplementary_Data [file qzaf062_supplementary_data.zip › Figure S6.pptx]

## Slide 1
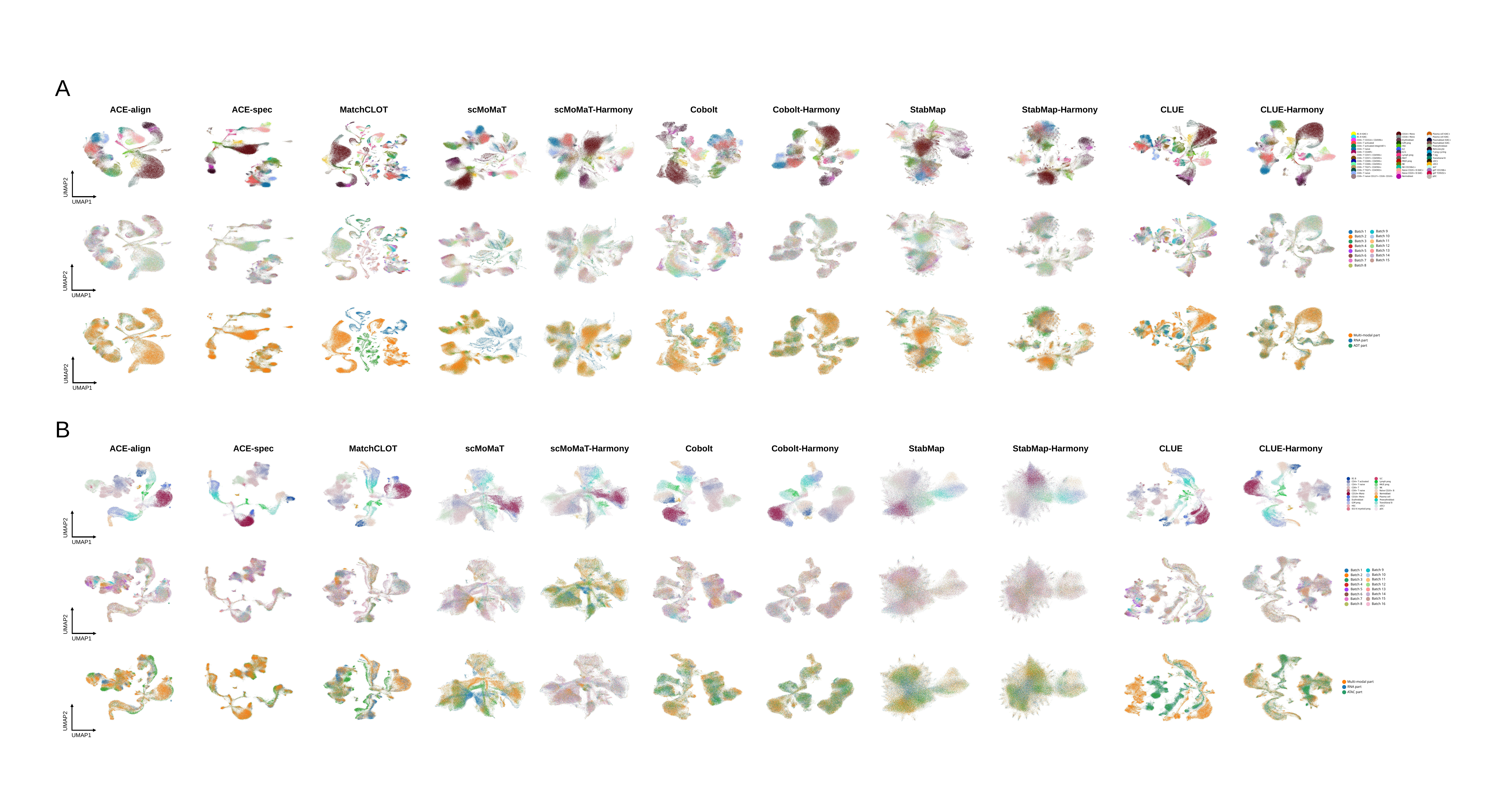

A
ACE-align
ACE-spec
MatchCLOT
scMoMaT
scMoMaT-Harmony
Cobolt
Cobolt-Harmony
StabMap
StabMap-Harmony
CLUE
CLUE-Harmony
UMAP2
UMAP1
Batch 9
Batch 1
Batch 10
Batch 2
Batch 11
Batch 3
Batch 12
Batch 4
Batch 13
Batch 5
Batch 14
Batch 6
UMAP2
UMAP1
Batch 15
Batch 7
Batch 8
Multi-modal part
RNA part
ADT part
UMAP2
UMAP1
B
ACE-align
ACE-spec
MatchCLOT
scMoMaT
scMoMaT-Harmony
Cobolt
Cobolt-Harmony
StabMap
StabMap-Harmony
CLUE
CLUE-Harmony
UMAP2
UMAP1
Batch 9
Batch 1
Batch 10
Batch 2
Batch 11
Batch 3
Batch 12
Batch 4
Batch 13
Batch 5
Batch 14
Batch 6
Batch 15
Batch 7
UMAP2
UMAP1
Batch 8
Batch 16
Multi-modal part
RNA part
ATAC part
UMAP2
UMAP1
