# Supplementary material for: ACE: A Versatile Contrastive Learning Framework for Single-cell Mosaic Integration
Source: Genomics Proteomics Bioinformatics. 2025 Aug 4;23(4):qzaf062. doi: 10.1093/gpbjnl/qzaf062 (PMC12582371; doi:10.1093/gpbjnl/qzaf062)
Supplement: qzaf062_Supplementary_Data [file qzaf062_supplementary_data.zip › Figure S7.pptx]

## Slide 1
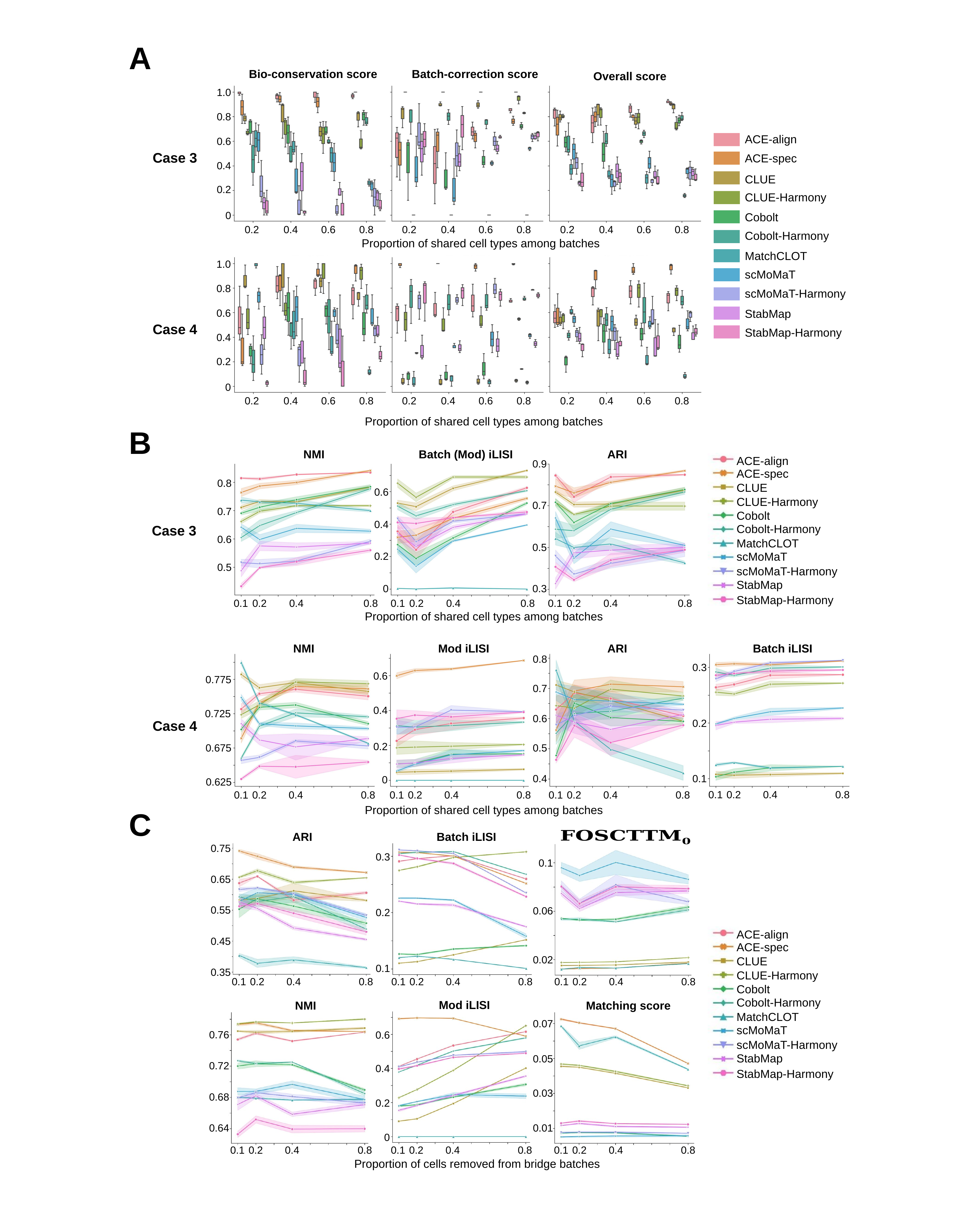

A
Bio-conservation score
Batch-correction score
Overall score
1.0
0.8
ACE-align
0.6
Case 3
ACE-spec
0.4
CLUE
0.2
CLUE-Harmony
0
Cobolt
0.2
0.4
0.6
0.8
0.2
0.4
0.6
0.8
0.2
0.4
0.6
0.8
Cobolt-Harmony
Proportion of shared cell types among batches
MatchCLOT
1.0
scMoMaT
0.8
scMoMaT-Harmony
StabMap
0.6
Case 4
StabMap-Harmony
0.4
0.2
0
0.2
0.4
0.6
0.8
0.2
0.4
0.6
0.8
0.2
0.4
0.6
0.8
Proportion of shared cell types among batches
B
Batch (Mod) iLISI
ARI
NMI
ACE-align
ACE-spec
CLUE
CLUE-Harmony
Cobolt
Cobolt-Harmony
MatchCLOT
scMoMaT
scMoMaT-Harmony
StabMap
StabMap-Harmony
0.9
0.1
0.2
0.4
0.8
0.8
0.6
0.7
0.7
0.4
Case 3
0.6
0.5
0.2
0.5
0
0.3
0.1
0.2
0.4
0.8
0.1
0.2
0.4
0.8
Proportion of shared cell types among batches
NMI
Mod iLISI
ARI
Batch iLISI
0.8
0.3
0.6
0.775
0.7
0.4
0.725
0.6
0.2
Case 4
0.2
0.5
0.675
0.4
0.1
0
0.625
0.1
0.2
0.4
0.8
0.1
0.2
0.4
0.8
0.1
0.2
0.4
0.8
0.1
0.2
0.4
0.8
Proportion of shared cell types among batches
C
ARI
0.75
0.65
0.55
0.45
0.35
0.1
0.2
0.4
0.8
Batch iLISI
0.3
0.2
0.1
0.1
0.2
0.4
0.8
0.1
0.06
0.02
0.1
0.2
0.4
0.8
ACE-align
ACE-spec
CLUE
CLUE-Harmony
Cobolt
Cobolt-Harmony
MatchCLOT
scMoMaT
scMoMaT-Harmony
StabMap
StabMap-Harmony
Mod iLISI
0.6
0.4
0.2
0.1
0.2
0.4
0.8
0
NMI
0.76
0.72
0.68
0.64
0.1
0.2
0.4
0.8
Matching score
0.07
0.05
0.03
0.01
0.1
0.2
0.4
0.8
Proportion of cells removed from bridge batches
