# Supplementary material for: ACE: A Versatile Contrastive Learning Framework for Single-cell Mosaic Integration
Source: Genomics Proteomics Bioinformatics. 2025 Aug 4;23(4):qzaf062. doi: 10.1093/gpbjnl/qzaf062 (PMC12582371; doi:10.1093/gpbjnl/qzaf062)
Supplement: qzaf062_Supplementary_Data [file qzaf062_supplementary_data.zip › Figure S9.pptx]

## Slide 1
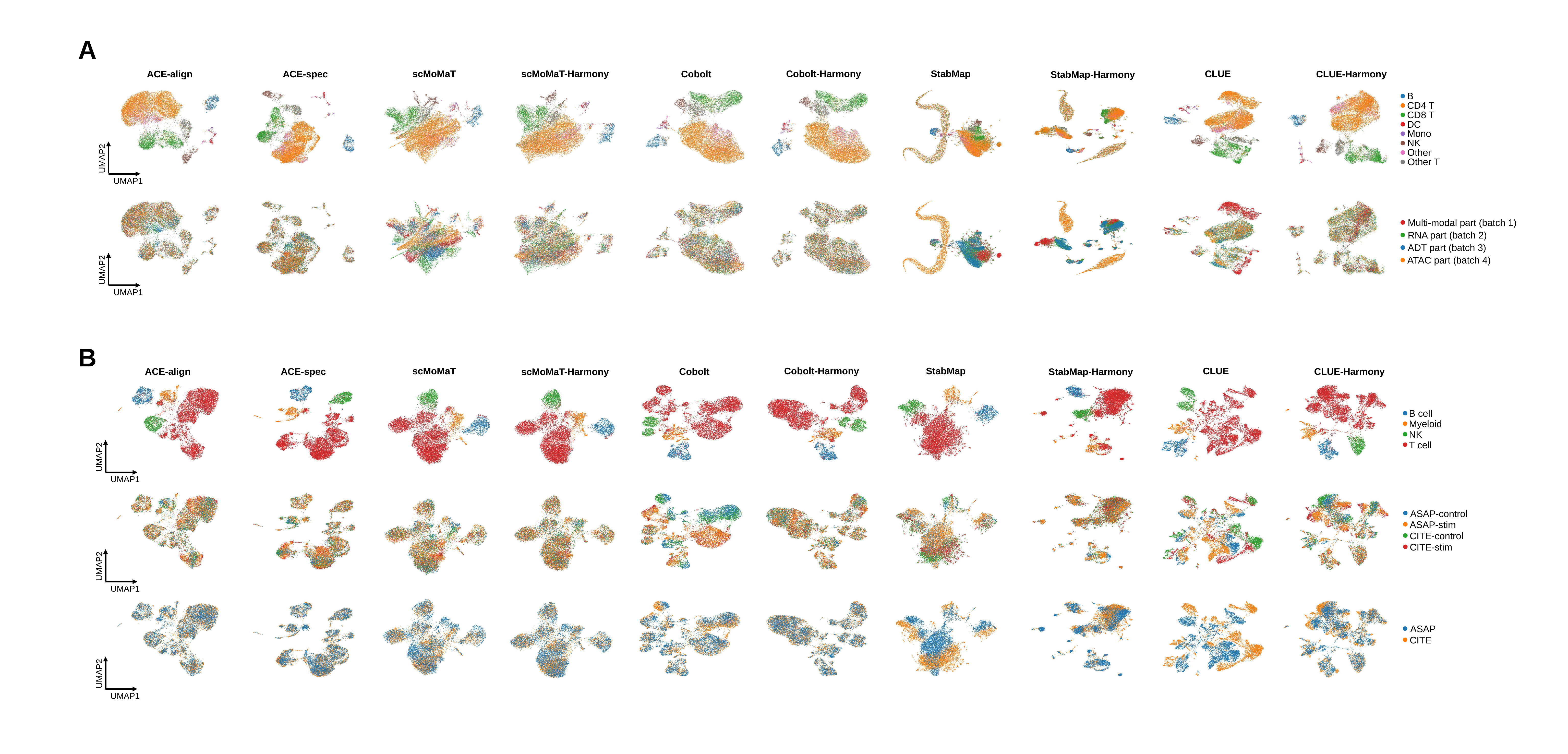

A
scMoMaT
scMoMaT-Harmony
Cobolt-Harmony
StabMap
CLUE
ACE-align
ACE-spec
Cobolt
CLUE-Harmony
StabMap-Harmony
B
CD4 T
CD8 T
DC
Mono
UMAP2
UMAP1
NK
Other
Other T
Multi-modal part (batch 1)
RNA part (batch 2)
UMAP2
UMAP1
ADT part (batch 3)
ATAC part (batch 4)
B
scMoMaT
Cobolt-Harmony
StabMap
CLUE
ACE-align
ACE-spec
Cobolt
CLUE-Harmony
scMoMaT-Harmony
StabMap-Harmony
B cell
Myeloid
UMAP2
UMAP1
NK
T cell
ASAP-control
ASAP-stim
CITE-control
UMAP2
UMAP1
CITE-stim
ASAP
CITE
UMAP2
UMAP1
