# Supplementary material for: ACE: A Versatile Contrastive Learning Framework for Single-cell Mosaic Integration
Source: Genomics Proteomics Bioinformatics. 2025 Aug 4;23(4):qzaf062. doi: 10.1093/gpbjnl/qzaf062 (PMC12582371; doi:10.1093/gpbjnl/qzaf062)
Supplement: qzaf062_Supplementary_Data [file qzaf062_supplementary_data.zip › Figure S10.pptx]

## Slide 1
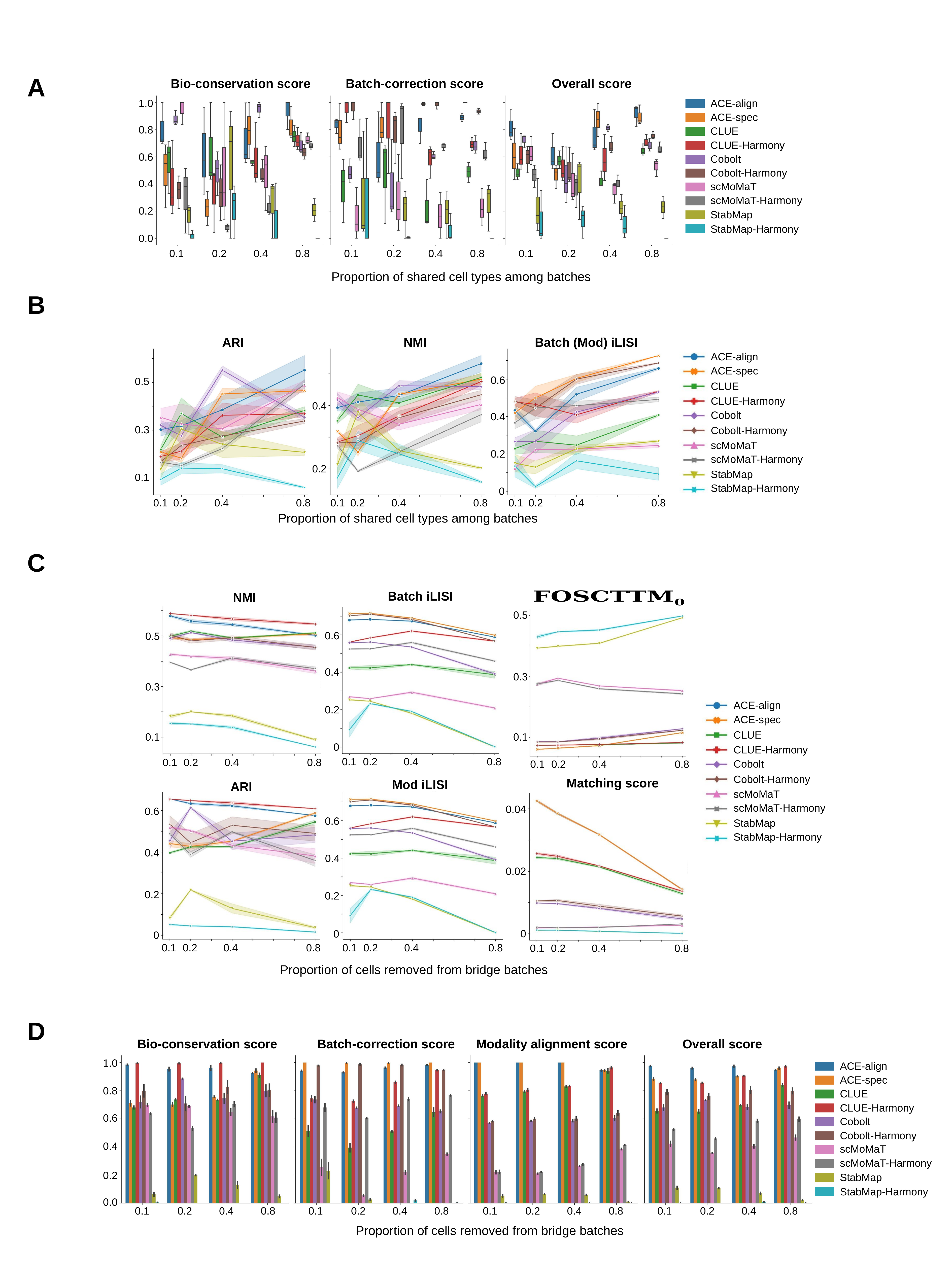

A
Bio-conservation score
Batch-correction score
Overall score
1.0
ACE-align
ACE-spec
CLUE
CLUE-Harmony
Cobolt
Cobolt-Harmony
scMoMaT
scMoMaT-Harmony
StabMap
StabMap-Harmony
0.8
0.6
0.4
0.2
0.0
0.1
0.2
0.4
0.8
0.1
0.2
0.4
0.8
0.1
0.2
0.4
0.8
Proportion of shared cell types among batches
B
ARI
NMI
Batch (Mod) iLISI
ACE-align
ACE-spec
CLUE
CLUE-Harmony
Cobolt
Cobolt-Harmony
scMoMaT
scMoMaT-Harmony
StabMap
StabMap-Harmony
0.6
0.5
0.4
0.4
0.3
0.2
0.2
0.1
0
0.1
0.2
0.4
0.8
0.1
0.2
0.4
0.8
0.1
0.2
0.4
0.8
Proportion of shared cell types among batches
C
Batch iLISI
0.6
0.4
0.2
0
0.1
0.2
0.4
0.8
NMI
0.5
0.3
0.1
0.1
0.2
0.4
0.8
0.5
0.3
0.1
0.1
0.2
0.4
0.8
ACE-align
ACE-spec
CLUE
CLUE-Harmony
Cobolt
Cobolt-Harmony
scMoMaT
scMoMaT-Harmony
StabMap
StabMap-Harmony
Matching score
0.04
0.02
0
0.1
0.2
0.4
0.8
Mod iLISI
0.6
0.4
0.2
0
0.1
0.2
0.4
0.8
ARI
0.6
0.4
0.2
0
0.1
0.2
0.4
0.8
Proportion of cells removed from bridge batches
D
Bio-conservation score
Batch-correction score
Modality alignment score
Overall score
1.0
0.8
0.6
0.4
0.2
0.0
0.1
0.2
0.4
0.8
0.1
0.2
0.4
0.8
0.1
0.2
0.4
0.8
0.1
0.2
0.4
0.8
Proportion of cells removed from bridge batches
ACE-align
ACE-spec
CLUE
CLUE-Harmony
Cobolt
Cobolt-Harmony
scMoMaT
scMoMaT-Harmony
StabMap
StabMap-Harmony
