# Supplementary material for: ACE: A Versatile Contrastive Learning Framework for Single-cell Mosaic Integration
Source: Genomics Proteomics Bioinformatics. 2025 Aug 4;23(4):qzaf062. doi: 10.1093/gpbjnl/qzaf062 (PMC12582371; doi:10.1093/gpbjnl/qzaf062)
Supplement: qzaf062_Supplementary_Data [file qzaf062_supplementary_data.zip › Figure S11.pptx]

## Slide 1
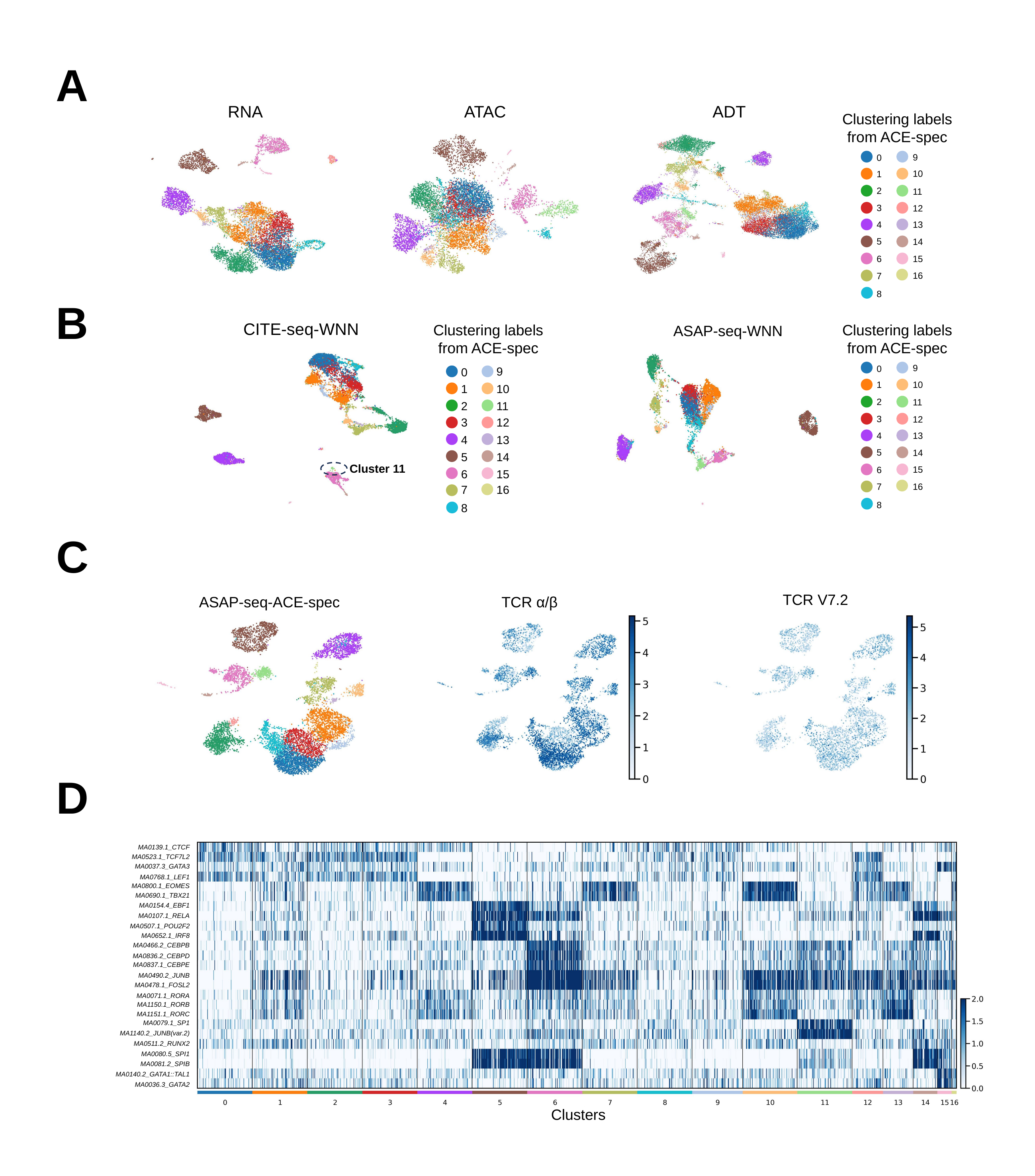

A
RNA
ATAC
ADT
Clustering labels from ACE-spec
9
0
10
1
2
11
3
12
13
4
5
14
15
6
16
7
8
B
CITE-seq-WNN
Clustering labels from ACE-spec
Clustering labels from ACE-spec
ASAP-seq-WNN
Cluster 11
9
0
10
1
2
11
3
12
13
4
5
14
15
6
16
7
8
9
0
1
10
2
11
3
12
4
13
5
14
6
15
7
16
8
C
TCR V7.2
ASAP-seq-ACE-spec
TCR α/β
D
MA0139.1_CTCF
MA0523.1_TCF7L2
MA0037.3_GATA3
MA0768.1_LEF1
MA0800.1_EOMES
MA0690.1_TBX21
MA0154.4_EBF1
MA0107.1_RELA
MA0507.1_POU2F2
MA0652.1_IRF8
MA0466.2_CEBPB
MA0836.2_CEBPD
MA0837.1_CEBPE
MA0490.2_JUNB
MA0478.1_FOSL2
MA0071.1_RORA
MA1150.1_RORB
MA1151.1_RORC
MA0079.1_SP1
MA1140.2_JUNB(var.2)
MA0511.2_RUNX2
MA0080.5_SPI1
MA0081.2_SPIB
MA0140.2_GATA1::TAL1
MA0036.3_GATA2
Clusters
