# Supplementary material for: ACE: A Versatile Contrastive Learning Framework for Single-cell Mosaic Integration
Source: Genomics Proteomics Bioinformatics. 2025 Aug 4;23(4):qzaf062. doi: 10.1093/gpbjnl/qzaf062 (PMC12582371; doi:10.1093/gpbjnl/qzaf062)
Supplement: qzaf062_Supplementary_Data [file qzaf062_supplementary_data.zip › Figure S12.pptx]

## Slide 1
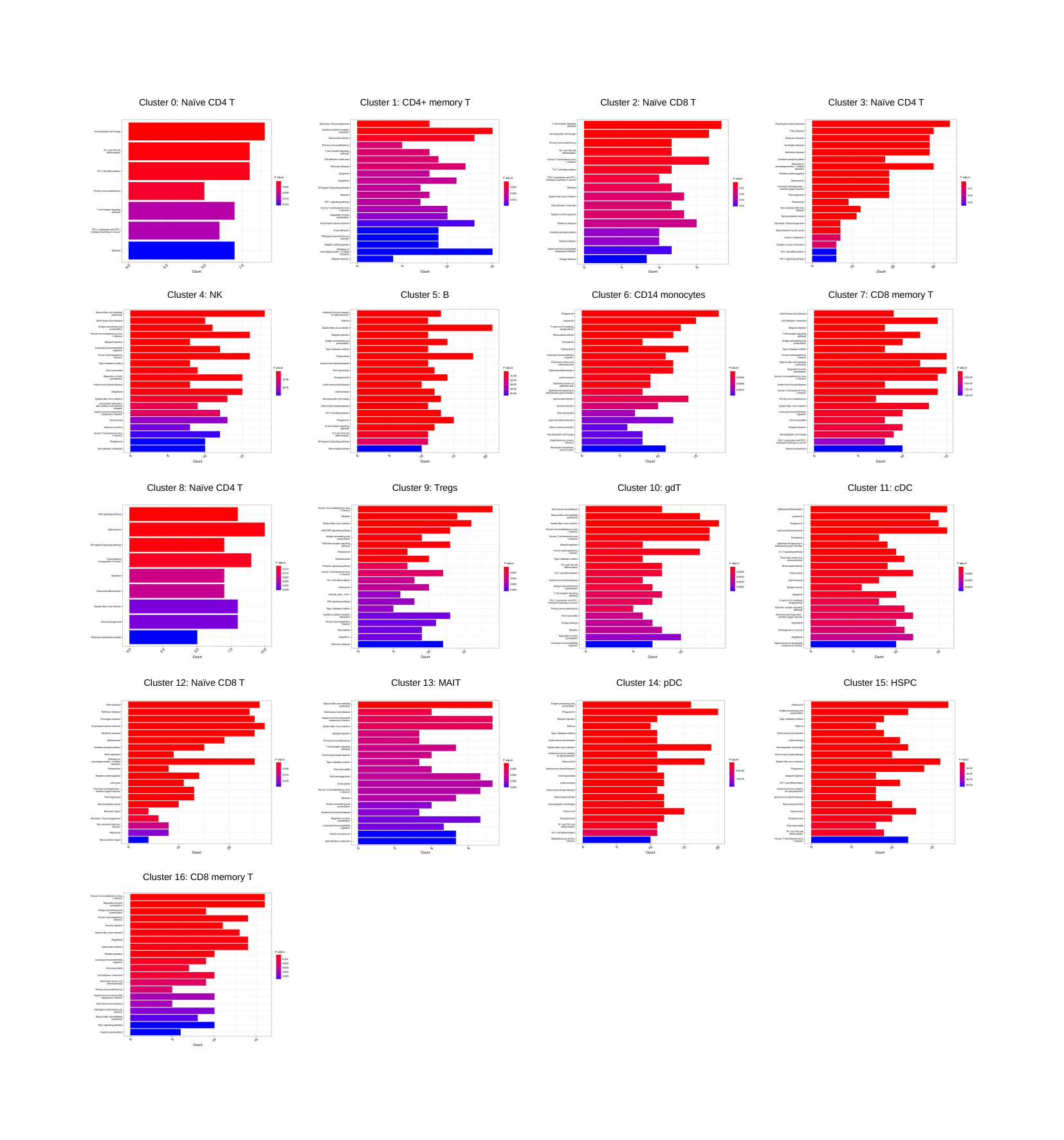

Cluster 0: Naïve CD4 T
Cluster 1: CD4+ memory T
Cluster 2: Naïve CD8 T
Cluster 3: Naïve CD4 T
Cluster 4: NK
Cluster 5: B
Cluster 6: CD14 monocytes
Cluster 7: CD8 memory T
Cluster 8: Naïve CD4 T
Cluster 9: Tregs
Cluster 10: gdT
Cluster 11: cDC
Cluster 12: Naïve CD8 T
Cluster 13: MAIT
Cluster 14: pDC
Cluster 15: HSPC
Cluster 16: CD8 memory T
P adjust
P adjust
P adjust
P adjust
P adjust
P adjust
P adjust
P adjust
P adjust
P adjust
P adjust
P adjust
P adjust
P adjust
P adjust
P adjust
P adjust
