# Supplementary material for: ACE: A Versatile Contrastive Learning Framework for Single-cell Mosaic Integration
Source: Genomics Proteomics Bioinformatics. 2025 Aug 4;23(4):qzaf062. doi: 10.1093/gpbjnl/qzaf062 (PMC12582371; doi:10.1093/gpbjnl/qzaf062)
Supplement: qzaf062_Supplementary_Data [file qzaf062_supplementary_data.zip › Figure S13.pptx]

## Slide 1
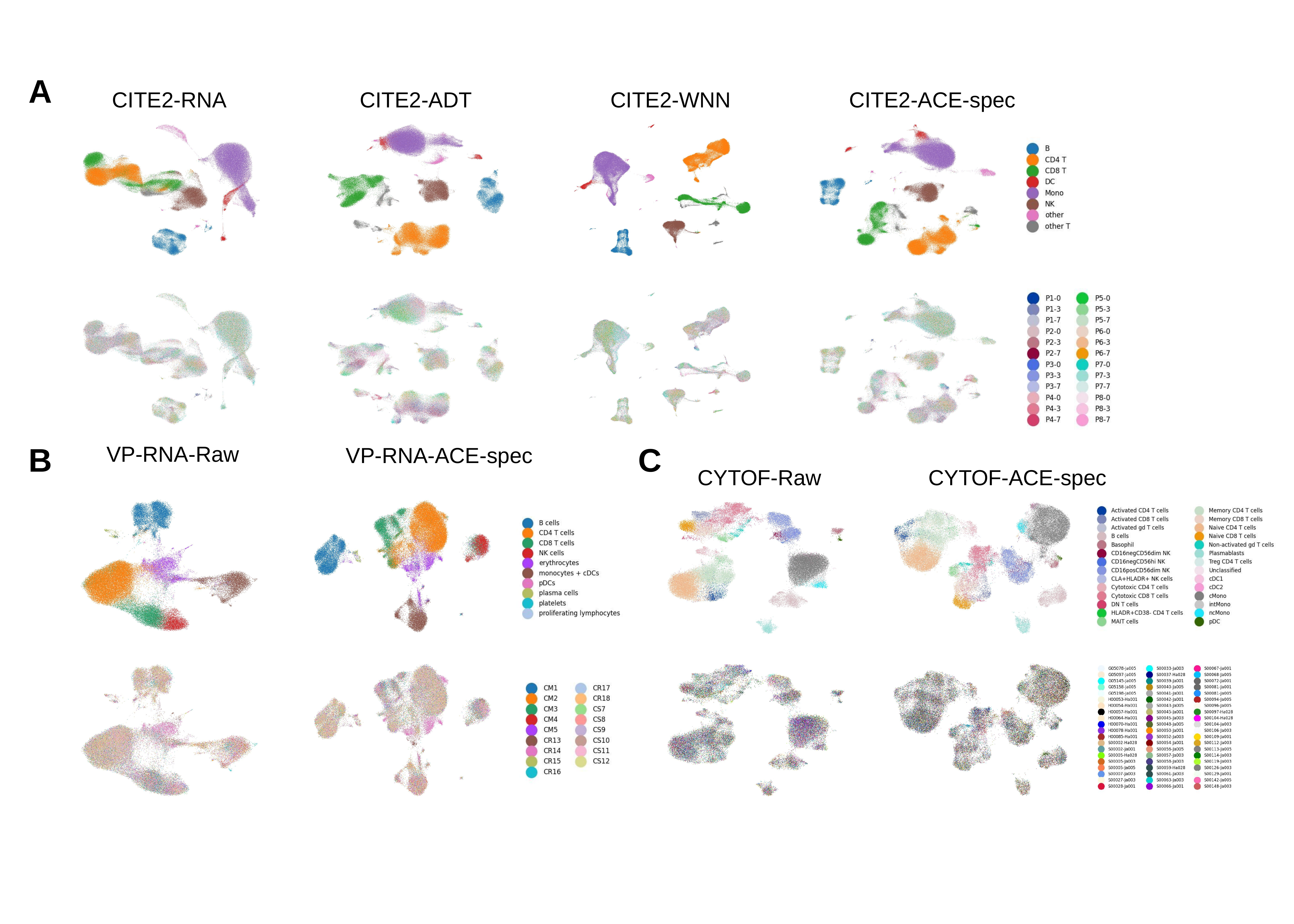

A
CITE2-RNA
CITE2-ADT
CITE2-WNN
CITE2-ACE-spec
B
C
VP-RNA-Raw
VP-RNA-ACE-spec
CYTOF-Raw
CYTOF-ACE-spec
