# Supplementary material for: ACE: A Versatile Contrastive Learning Framework for Single-cell Mosaic Integration
Source: Genomics Proteomics Bioinformatics. 2025 Aug 4;23(4):qzaf062. doi: 10.1093/gpbjnl/qzaf062 (PMC12582371; doi:10.1093/gpbjnl/qzaf062)
Supplement: qzaf062_Supplementary_Data [file qzaf062_supplementary_data.zip › Figure S14.pptx]

## Slide 1
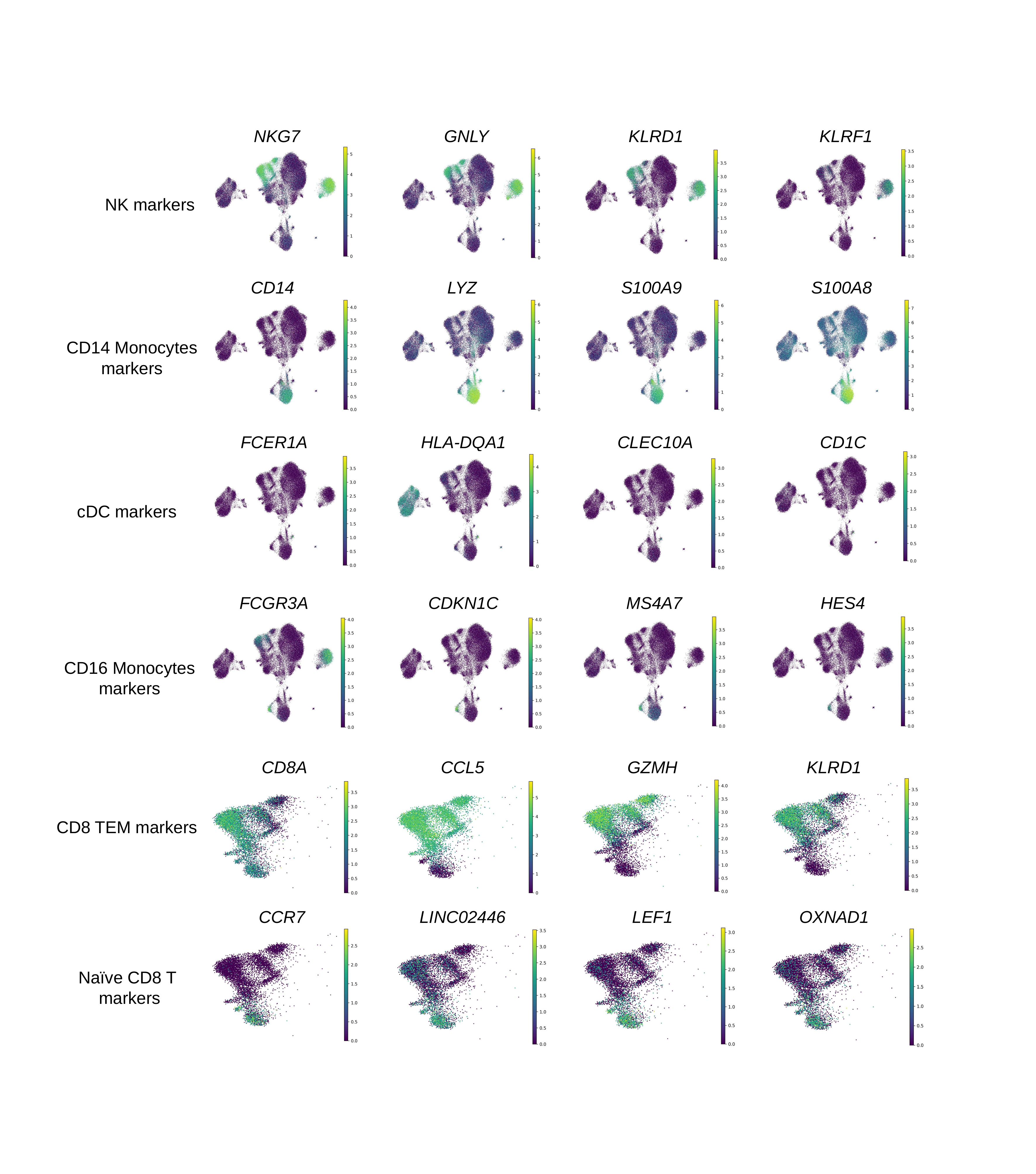

NKG7
GNLY
KLRD1
KLRF1
NK markers
CD14
LYZ
S100A9
S100A8
CD14 Monocytes markers
FCER1A
HLA-DQA1
CLEC10A
CD1C
cDC markers
FCGR3A
CDKN1C
MS4A7
HES4
CD16 Monocytes markers
CD8A
CCL5
GZMH
KLRD1
CD8 TEM markers
CCR7
LINC02446
LEF1
OXNAD1
Naïve CD8 T
markers
