# Supplementary material for: ACE: A Versatile Contrastive Learning Framework for Single-cell Mosaic Integration
Source: Genomics Proteomics Bioinformatics. 2025 Aug 4;23(4):qzaf062. doi: 10.1093/gpbjnl/qzaf062 (PMC12582371; doi:10.1093/gpbjnl/qzaf062)
Supplement: qzaf062_Supplementary_Data [file qzaf062_supplementary_data.zip › Figure S15.pptx]

## Slide 1
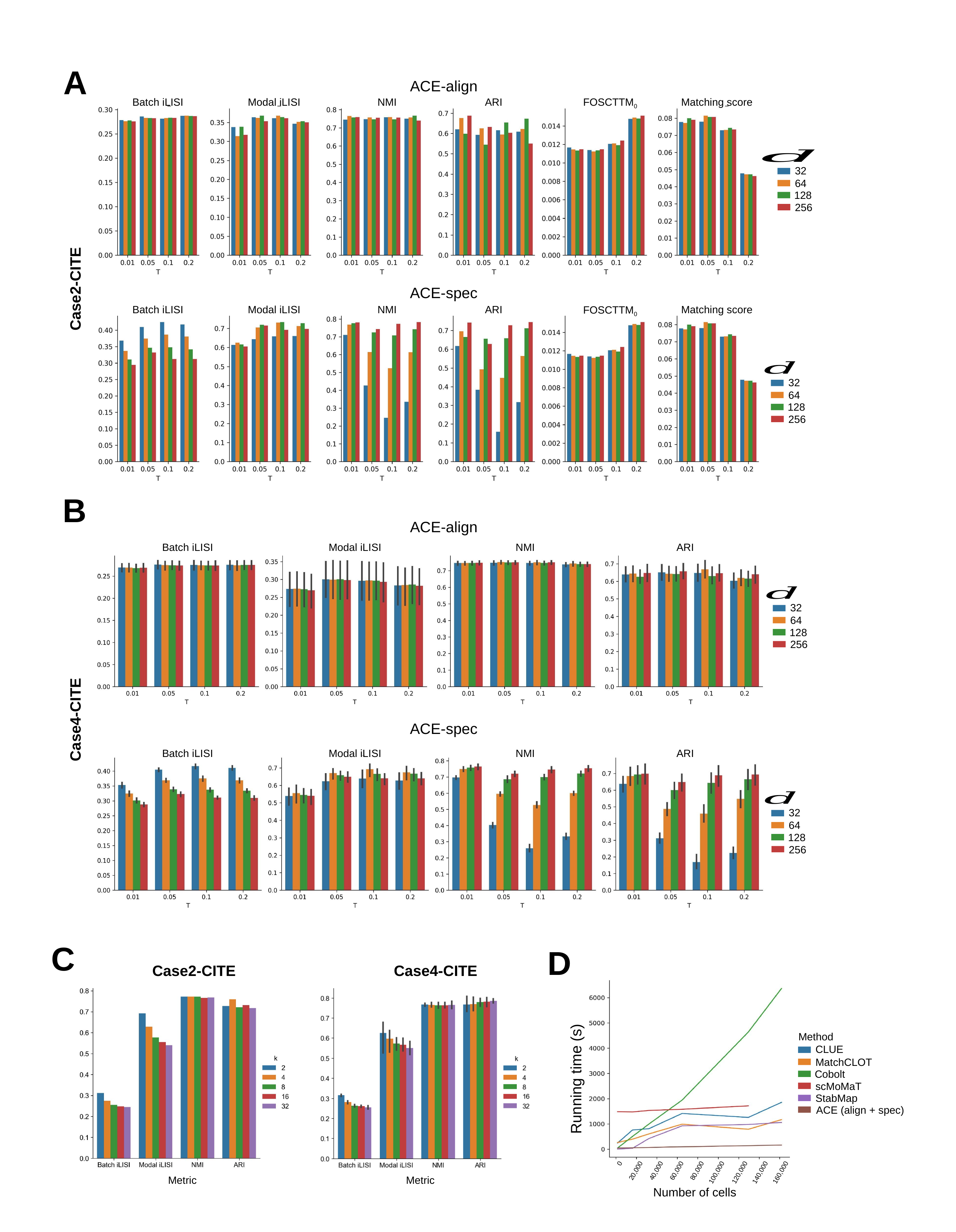

A
ACE-align
Batch iLISI
Modal iLISI
NMI
ARI
FOSCTTM0
Matching score
32
64
128
256
Case2-CITE
ACE-spec
Batch iLISI
Modal iLISI
NMI
ARI
Matching score
FOSCTTM0
32
64
128
256
B
ACE-align
Batch iLISI
Modal iLISI
NMI
ARI
32
64
128
256
Case4-CITE
ACE-spec
Batch iLISI
Modal iLISI
NMI
ARI
32
64
128
256
C
D
Case2-CITE
Case4-CITE
Metric
Metric
Method
CLUE
MatchCLOT
Cobolt
Running time (s)
scMoMaT
StabMap
ACE (align + spec)
Number of cells
0
20,000
40,000
60,000
80,000
100,000
120,000
140,000
160,000
