# Supplementary material for: ACE: A Versatile Contrastive Learning Framework for Single-cell Mosaic Integration
Source: Genomics Proteomics Bioinformatics. 2025 Aug 4;23(4):qzaf062. doi: 10.1093/gpbjnl/qzaf062 (PMC12582371; doi:10.1093/gpbjnl/qzaf062)
Supplement: qzaf062_Supplementary_Data [file qzaf062_supplementary_data.zip › Figure S16.pptx]

## Slide 1
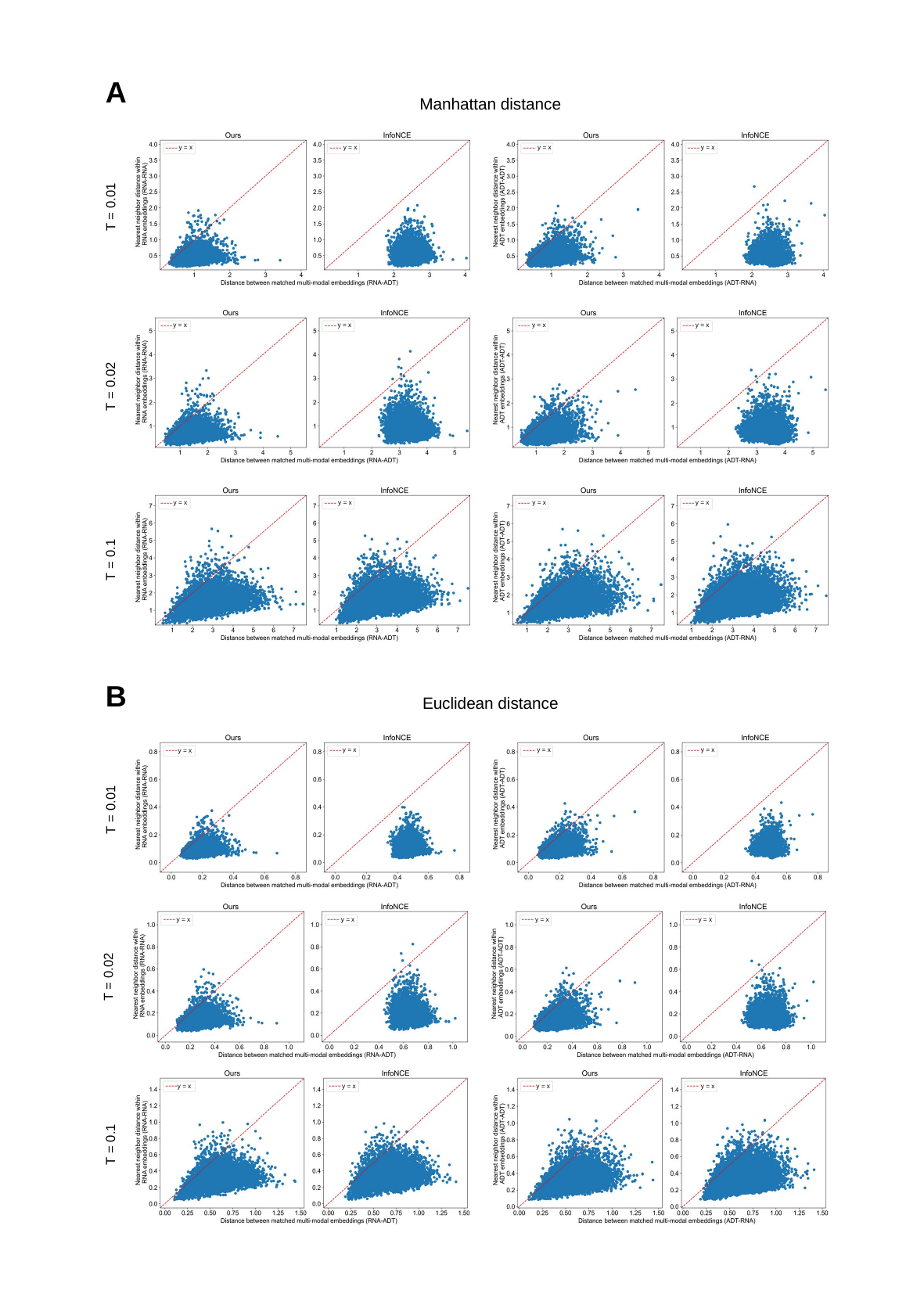

A
Manhattan distance
T = 0.01
T = 0.02
T = 0.1
B
Euclidean distance
T = 0.01
T = 0.02
T = 0.1
y = x
y = x
y = x
y = x
y = x
y = x
y = x
y = x
y = x
y = x
y = x
y = x
y = x
y = x
y = x
y = x
y = x
y = x
y = x
y = x
y = x
y = x
y = x
y = x
