# Supplementary material for: ACE: A Versatile Contrastive Learning Framework for Single-cell Mosaic Integration
Source: Genomics Proteomics Bioinformatics. 2025 Aug 4;23(4):qzaf062. doi: 10.1093/gpbjnl/qzaf062 (PMC12582371; doi:10.1093/gpbjnl/qzaf062)
Supplement: qzaf062_Supplementary_Data [file qzaf062_supplementary_data.zip › Figure S17.pptx]

## Slide 1
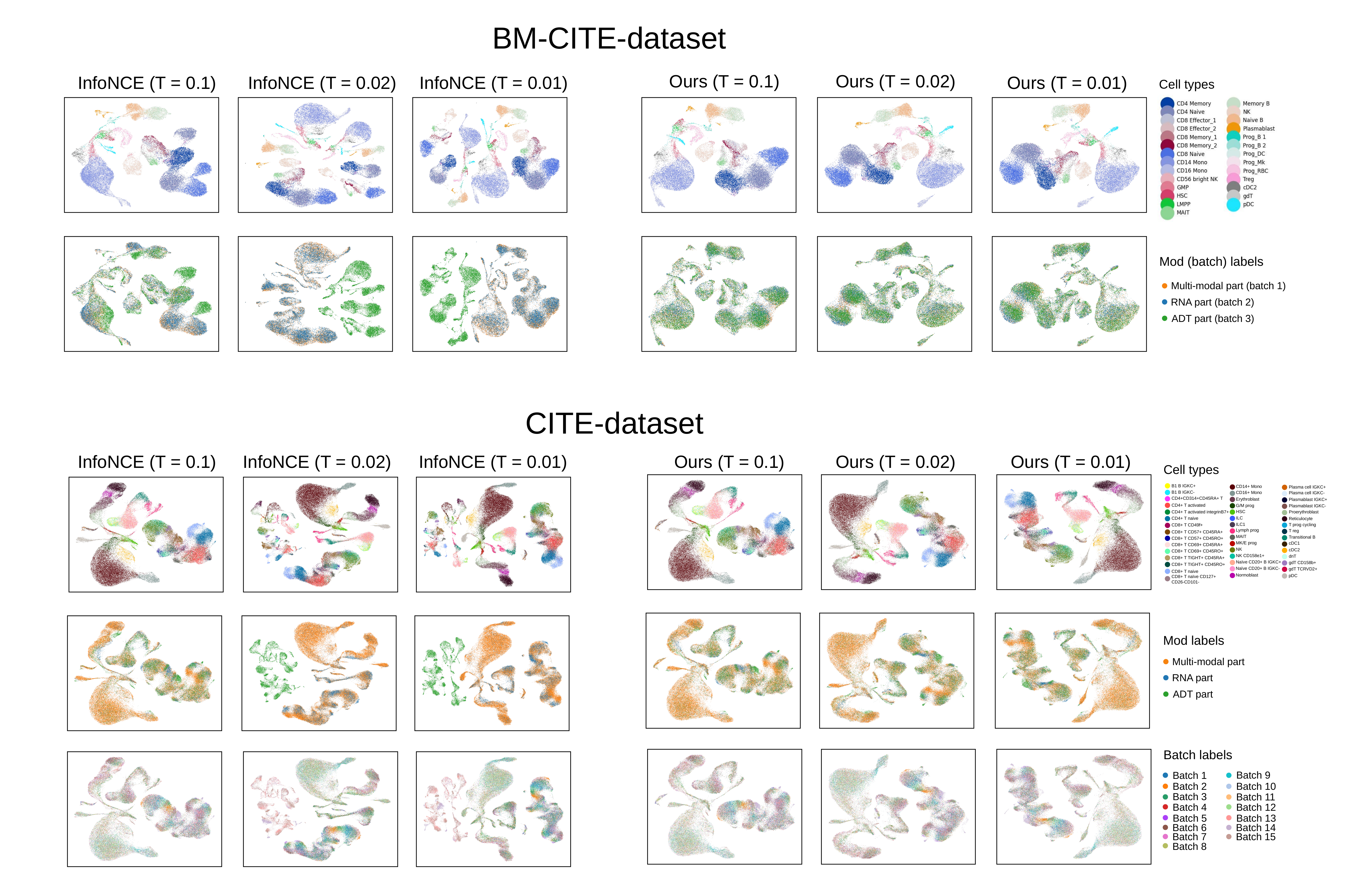

BM-CITE-dataset
Ours (T = 0.1)
Ours (T = 0.02)
Ours (T = 0.01)
InfoNCE (T = 0.1)
InfoNCE (T = 0.02)
InfoNCE (T = 0.01)
Cell types
Mod (batch) labels
Multi-modal part (batch 1)
RNA part (batch 2)
ADT part (batch 3)
CITE-dataset
InfoNCE (T = 0.1)
InfoNCE (T = 0.02)
InfoNCE (T = 0.01)
Ours (T = 0.1)
Ours (T = 0.02)
Ours (T = 0.01)
Cell types
B1 B IGKC+
CD14+ Mono
Plasma cell IGKC+
B1 B IGKC-
CD16+ Mono
Plasma cell IGKC-
CD4+CD314+CD45RA+ T
Erythroblast
Plasmablast IGKC+
CD4+ T activated
G/M prog
Plasmablast IGKC-
HSC
CD4+ T activated integrinB7+
Proerythroblast
ILC
CD4+ T naive
Reticulocyte
ILC1
T prog cycling
CD8+ T CD49f+
Lymph prog
T reg
CD8+ T CD57+ CD45RA+
MAIT
Transitional B
CD8+ T CD57+ CD45RO+
MK/E prog
cDC1
CD8+ T CD69+ CD45RA+
NK
cDC2
CD8+ T CD69+ CD45RO+
NK CD158e1+
dnT
CD8+ T TIGHT+ CD45RA+
Naïve CD20+ B IGKC+
gdT CD158b+
CD8+ T TIGHT+ CD45RO+
Naïve CD20+ B IGKC-
gdT TCRVD2+
CD8+ T naive
Normoblast
pDC
CD8+ T naïve CD127+ CD26-CD101-
Mod labels
Multi-modal part
RNA part
ADT part
Batch labels
Batch 9
Batch 10
Batch 11
Batch 12
Batch 13
Batch 14
Batch 15
Batch 1
Batch 2
Batch 3
Batch 4
Batch 5
Batch 6
Batch 7
Batch 8
