# Supplementary material for: ACE: A Versatile Contrastive Learning Framework for Single-cell Mosaic Integration
Source: Genomics Proteomics Bioinformatics. 2025 Aug 4;23(4):qzaf062. doi: 10.1093/gpbjnl/qzaf062 (PMC12582371; doi:10.1093/gpbjnl/qzaf062)
Supplement: qzaf062_Supplementary_Data [file qzaf062_supplementary_data.zip › Figure S25.pptx]

## Slide 1
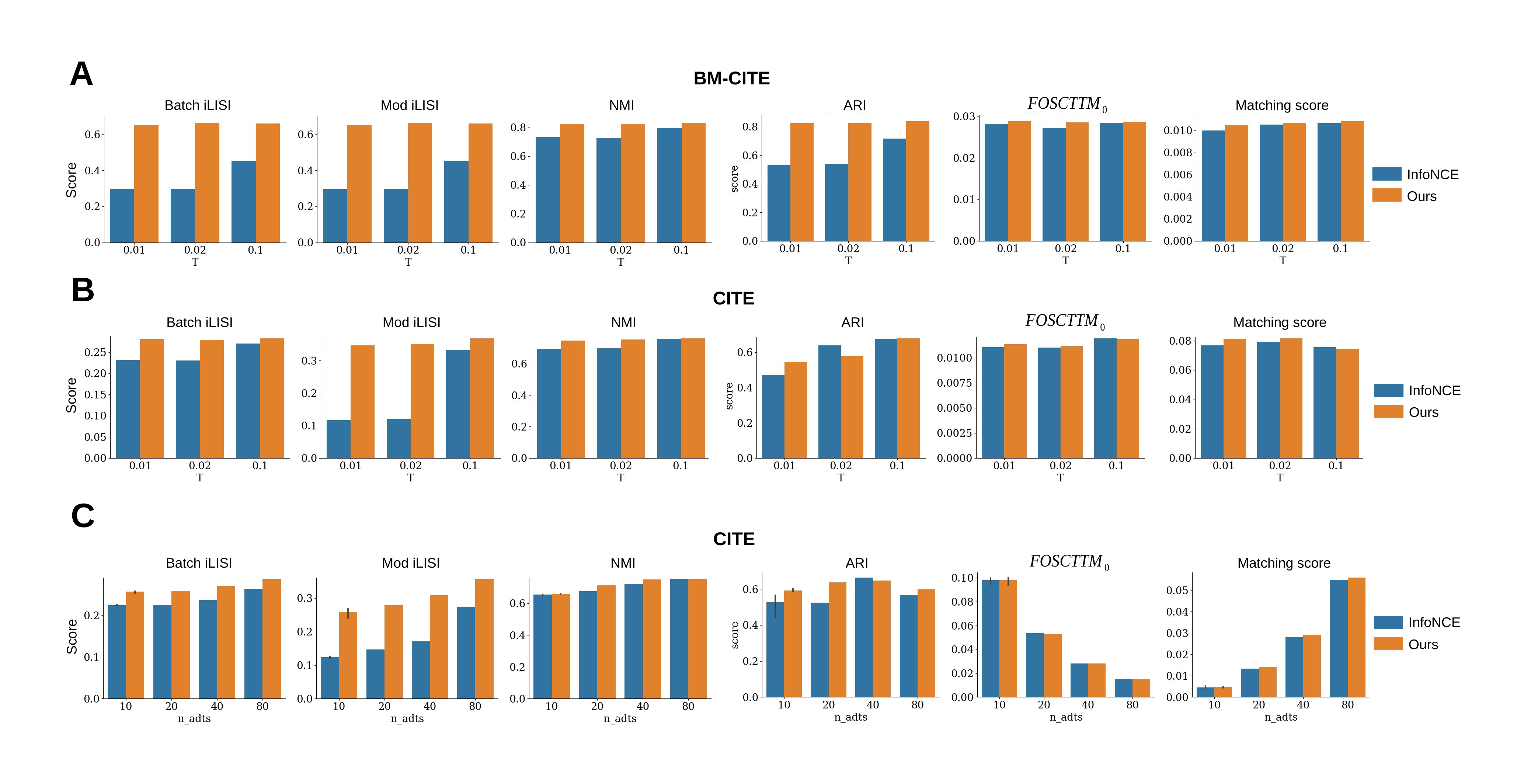

A
BM-CITE
Batch iLISI
Mod iLISI
NMI
ARI
Matching score
Score
InfoNCE
Ours
B
CITE
Batch iLISI
Mod iLISI
NMI
ARI
Matching score
Score
InfoNCE
Ours
C
CITE
Batch iLISI
Mod iLISI
NMI
ARI
Matching score
InfoNCE
Ours
Score
