# Supplementary material for: ACE: A Versatile Contrastive Learning Framework for Single-cell Mosaic Integration
Source: Genomics Proteomics Bioinformatics. 2025 Aug 4;23(4):qzaf062. doi: 10.1093/gpbjnl/qzaf062 (PMC12582371; doi:10.1093/gpbjnl/qzaf062)
Supplement: qzaf062_Supplementary_Data [file qzaf062_supplementary_data.zip › Figure S28.pptx]

## Slide 1
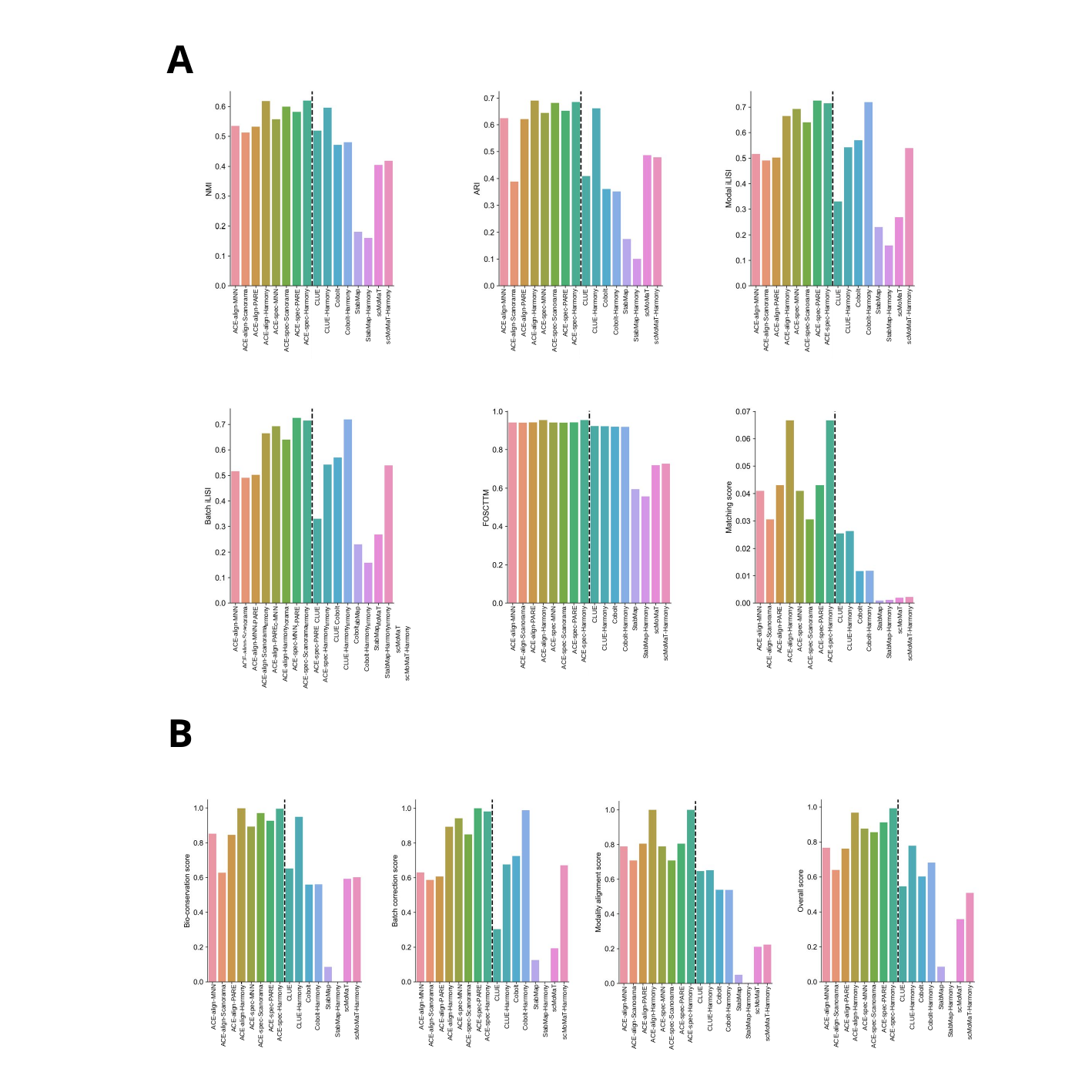

A
ACE-spec-MNN
StabMap
ACE-align-MNN
CLUE
ACE-spec-Harmony
scMoMaT-Harmony
ACE-align-Scanorama
ACE-align-PARE
ACE-align-Harmony
ACE-spec-Scanorama
ACE-spec-PARE
CLUE-Harmony
Cobolt
Cobolt-Harmony
StabMap-Harmony
scMoMaT
ACE-spec-MNN
StabMap
ACE-align-MNN
CLUE
ACE-spec-Harmony
scMoMaT-Harmony
ACE-align-Scanorama
ACE-align-PARE
ACE-align-Harmony
ACE-spec-Scanorama
ACE-spec-PARE
CLUE-Harmony
Cobolt
Cobolt-Harmony
StabMap-Harmony
scMoMaT
ACE-spec-MNN
StabMap
ACE-align-MNN
CLUE
ACE-spec-Harmony
scMoMaT-Harmony
ACE-align-Scanorama
ACE-align-PARE
ACE-align-Harmony
ACE-spec-Scanorama
ACE-spec-PARE
CLUE-Harmony
Cobolt
Cobolt-Harmony
StabMap-Harmony
scMoMaT
ACE-spec-MNN
StabMap
ACE-align-MNN
CLUE
ACE-spec-Harmony
scMoMaT-Harmony
ACE-align-Scanorama
ACE-align-PARE
ACE-align-Harmony
ACE-spec-Scanorama
ACE-spec-PARE
CLUE-Harmony
Cobolt
Cobolt-Harmony
StabMap-Harmony
scMoMaT
ACE-spec-MNN
StabMap
ACE-align-MNN
CLUE
ACE-spec-Harmony
scMoMaT-Harmony
ACE-align-Scanorama
ACE-align-PARE
ACE-align-Harmony
ACE-spec-Scanorama
ACE-spec-PARE
CLUE-Harmony
Cobolt
Cobolt-Harmony
StabMap-Harmony
scMoMaT
ACE-spec-MNN
StabMap
ACE-align-MNN
CLUE
ACE-spec-Harmony
scMoMaT-Harmony
ACE-align-Scanorama
ACE-align-PARE
ACE-align-Harmony
ACE-spec-Scanorama
ACE-spec-PARE
CLUE-Harmony
Cobolt
Cobolt-Harmony
StabMap-Harmony
scMoMaT
ACE-spec-MNN
StabMap
ACE-align-MNN
CLUE
ACE-spec-Harmony
scMoMaT-Harmony
ACE-align-Scanorama
ACE-align-PARE
ACE-align-Harmony
ACE-spec-Scanorama
ACE-spec-PARE
CLUE-Harmony
Cobolt
Cobolt-Harmony
StabMap-Harmony
scMoMaT
B
ACE-spec-MNN
StabMap
ACE-align-MNN
CLUE
ACE-spec-Harmony
scMoMaT-Harmony
ACE-align-Scanorama
ACE-align-PARE
ACE-align-Harmony
ACE-spec-Scanorama
ACE-spec-PARE
CLUE-Harmony
Cobolt
Cobolt-Harmony
StabMap-Harmony
scMoMaT
ACE-spec-MNN
StabMap
ACE-align-MNN
CLUE
ACE-spec-Harmony
scMoMaT-Harmony
ACE-align-Scanorama
ACE-align-PARE
ACE-align-Harmony
ACE-spec-Scanorama
ACE-spec-PARE
CLUE-Harmony
Cobolt
Cobolt-Harmony
StabMap-Harmony
scMoMaT
ACE-spec-MNN
StabMap
ACE-align-MNN
CLUE
ACE-spec-Harmony
scMoMaT-Harmony
ACE-align-Scanorama
ACE-align-PARE
ACE-align-Harmony
ACE-spec-Scanorama
ACE-spec-PARE
CLUE-Harmony
Cobolt
Cobolt-Harmony
StabMap-Harmony
scMoMaT
ACE-spec-MNN
StabMap
ACE-align-MNN
CLUE
ACE-spec-Harmony
scMoMaT-Harmony
ACE-align-Scanorama
ACE-align-PARE
ACE-align-Harmony
ACE-spec-Scanorama
ACE-spec-PARE
CLUE-Harmony
Cobolt
Cobolt-Harmony
StabMap-Harmony
scMoMaT
