# Supplementary material for: ACE: A Versatile Contrastive Learning Framework for Single-cell Mosaic Integration
Source: Genomics Proteomics Bioinformatics. 2025 Aug 4;23(4):qzaf062. doi: 10.1093/gpbjnl/qzaf062 (PMC12582371; doi:10.1093/gpbjnl/qzaf062)
Supplement: qzaf062_Supplementary_Data [file qzaf062_supplementary_data.zip › Figure S31.pptx]

## Slide 1
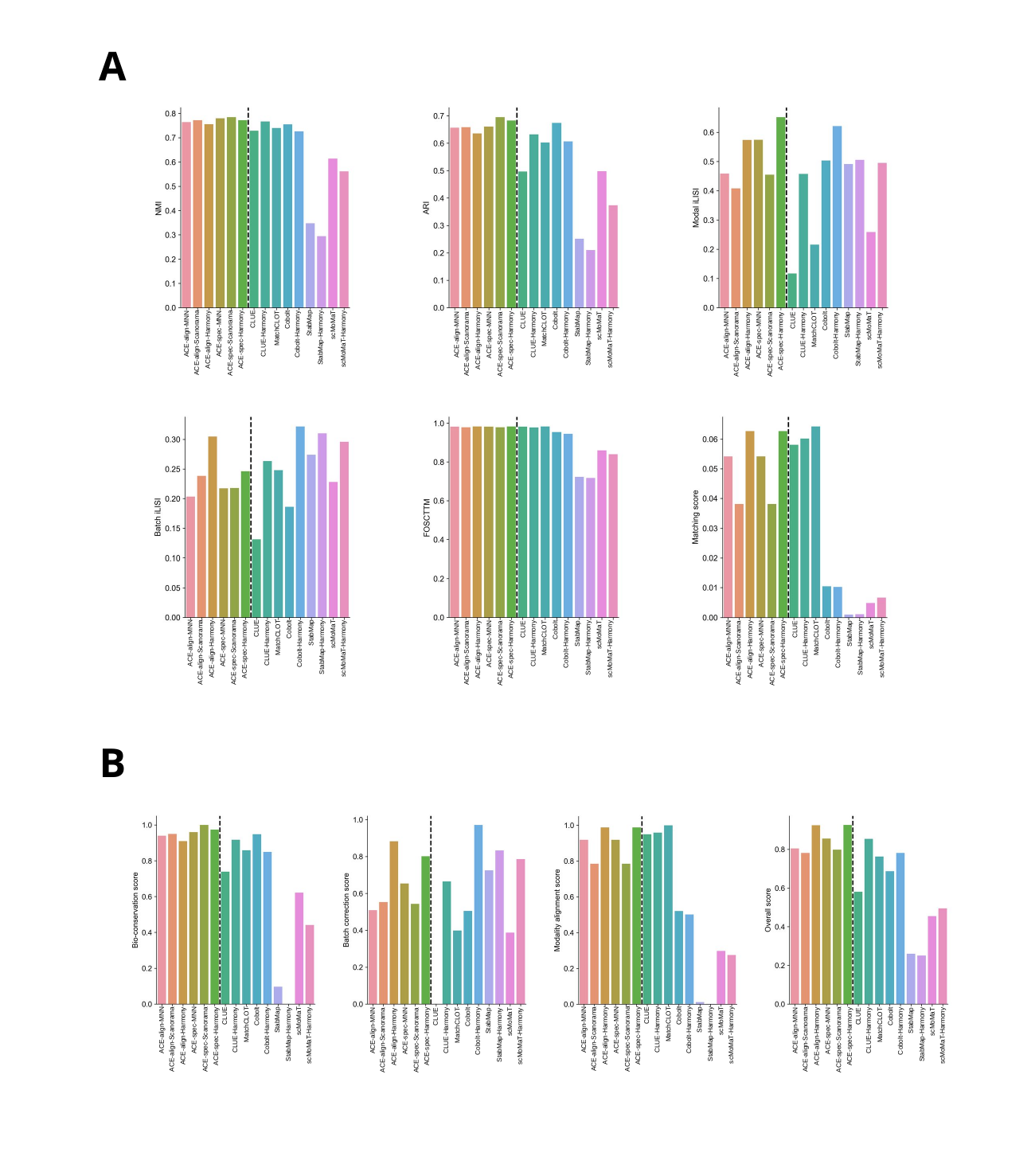

A
MatchCLOT
StabMap-Harmony
ACE-align-MNN
MatchCLOT
ACE-spec-Scanorama
CLUE-Harmony
scMoMaT-Harmony
ACE-align-Scanorama
ACE-align-Harmony
ACE-spec-MNN
ACE-spec-Harmony
CLUE
Cobolt
Cobolt-Harmony
StabMap
scMoMaT
MatchCLOT
StabMap-Harmony
ACE-align-MNN
MatchCLOT
ACE-spec-Scanorama
CLUE-Harmony
scMoMaT-Harmony
ACE-align-Scanorama
ACE-align-Harmony
ACE-spec-MNN
ACE-spec-Harmony
CLUE
Cobolt
Cobolt-Harmony
StabMap
scMoMaT
Cobolt
CLUE-Harmony
Cobolt-Harmony
StabMap-Harmony
ACE-align-MNN
MatchCLOT
ACE-spec-Scanorama
scMoMaT-Harmony
ACE-align-Scanorama
ACE-align-Harmony
ACE-spec-MNN
ACE-spec-Harmony
CLUE
StabMap
scMoMaT
MatchCLOT
StabMap-Harmony
ACE-align-MNN
MatchCLOT
ACE-spec-Scanorama
CLUE-Harmony
scMoMaT-Harmony
ACE-align-Scanorama
ACE-align-Harmony
ACE-spec-MNN
ACE-spec-Harmony
CLUE
Cobolt
Cobolt-Harmony
StabMap
scMoMaT
MatchCLOT
StabMap-Harmony
ACE-align-MNN
MatchCLOT
ACE-spec-Scanorama
CLUE-Harmony
scMoMaT-Harmony
ACE-align-Scanorama
ACE-align-Harmony
ACE-spec-MNN
ACE-spec-Harmony
CLUE
Cobolt
Cobolt-Harmony
StabMap
scMoMaT
MatchCLOT
StabMap-Harmony
ACE-align-MNN
MatchCLOT
ACE-spec-Scanorama
CLUE-Harmony
scMoMaT-Harmony
ACE-align-Scanorama
ACE-align-Harmony
ACE-spec-MNN
ACE-spec-Harmony
CLUE
Cobolt
Cobolt-Harmony
StabMap
scMoMaT
B
MatchCLOT
StabMap-Harmony
ACE-align-MNN
MatchCLOT
ACE-spec-Scanorama
CLUE-Harmony
scMoMaT-Harmony
ACE-align-Scanorama
ACE-align-Harmony
ACE-spec-MNN
ACE-spec-Harmony
Cobolt
Cobolt-Harmony
scMoMaT
CLUE
StabMap
MatchCLOT
StabMap-Harmony
ACE-align-MNN
MatchCLOT
ACE-spec-Scanorama
CLUE-Harmony
scMoMaT-Harmony
ACE-align-Scanorama
ACE-align-Harmony
ACE-spec-MNN
ACE-spec-Harmony
Cobolt
Cobolt-Harmony
scMoMaT
CLUE
StabMap
MatchCLOT
StabMap-Harmony
ACE-align-MNN
MatchCLOT
ACE-spec-Scanorama
CLUE-Harmony
scMoMaT-Harmony
ACE-align-Scanorama
ACE-align-Harmony
ACE-spec-MNN
ACE-spec-Harmony
Cobolt
Cobolt-Harmony
scMoMaT
CLUE
StabMap
MatchCLOT
StabMap-Harmony
ACE-align-MNN
MatchCLOT
ACE-spec-Scanorama
CLUE-Harmony
scMoMaT-Harmony
ACE-align-Scanorama
ACE-align-Harmony
ACE-spec-MNN
ACE-spec-Harmony
Cobolt
Cobolt-Harmony
scMoMaT
CLUE
StabMap
