# Supplementary material for: ACE: A Versatile Contrastive Learning Framework for Single-cell Mosaic Integration
Source: Genomics Proteomics Bioinformatics. 2025 Aug 4;23(4):qzaf062. doi: 10.1093/gpbjnl/qzaf062 (PMC12582371; doi:10.1093/gpbjnl/qzaf062)
Supplement: qzaf062_Supplementary_Data [file qzaf062_supplementary_data.zip › Figure S33.pptx]

## Slide 1
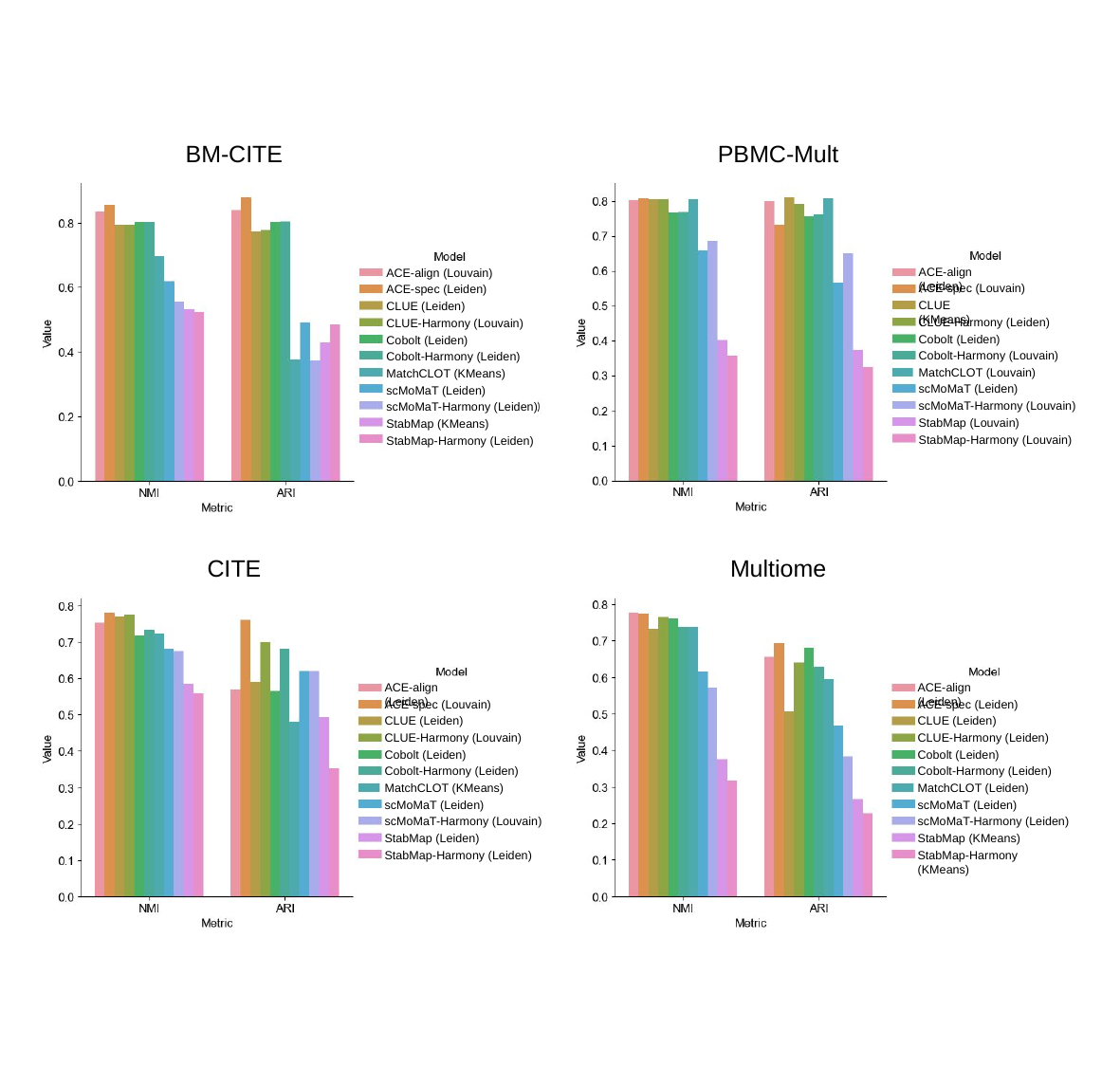

BM-CITE
PBMC-Mult
CITE
Multiome
ACE-align (Leiden)
ACE-spec (Louvain)
CLUE (KMeans)
CLUE-Harmony (Leiden)
Cobolt (Leiden)
Cobolt-Harmony (Louvain)
MatchCLOT (Louvain)
scMoMaT (Leiden)
StabMap (Louvain)
StabMap-Harmony (Louvain)
scMoMaT-Harmony (Louvain)
ACE-align (Louvain)
ACE-spec (Leiden)
CLUE (Leiden)
CLUE-Harmony (Louvain)
Cobolt (Leiden)
Cobolt-Harmony (Leiden)
MatchCLOT (KMeans)
scMoMaT (Leiden)
StabMap (KMeans)
StabMap-Harmony (Leiden)
scMoMaT-Harmony (Leiden)
MatchCLOT
ACE-align (Leiden)
ACE-spec (Louvain)
CLUE (Leiden)
CLUE-Harmony (Louvain)
Cobolt (Leiden)
Cobolt-Harmony (Leiden)
MatchCLOT (KMeans)
scMoMaT (Leiden)
StabMap (Leiden)
StabMap-Harmony (Leiden)
scMoMaT-Harmony (Louvain)
ACE-align (Leiden)
ACE-spec (Leiden)
CLUE (Leiden)
CLUE-Harmony (Leiden)
Cobolt (Leiden)
Cobolt-Harmony (Leiden)
MatchCLOT (Leiden)
scMoMaT (Leiden)
StabMap (KMeans)
StabMap-Harmony (KMeans)
scMoMaT-Harmony (Leiden)
MatchCLOT
MatchCLOT
