# Supplementary material for: ACE: A Versatile Contrastive Learning Framework for Single-cell Mosaic Integration
Source: Genomics Proteomics Bioinformatics. 2025 Aug 4;23(4):qzaf062. doi: 10.1093/gpbjnl/qzaf062 (PMC12582371; doi:10.1093/gpbjnl/qzaf062)
Supplement: qzaf062_Supplementary_Data [file qzaf062_supplementary_data.zip › Figure S36.pptx]

## Slide 1
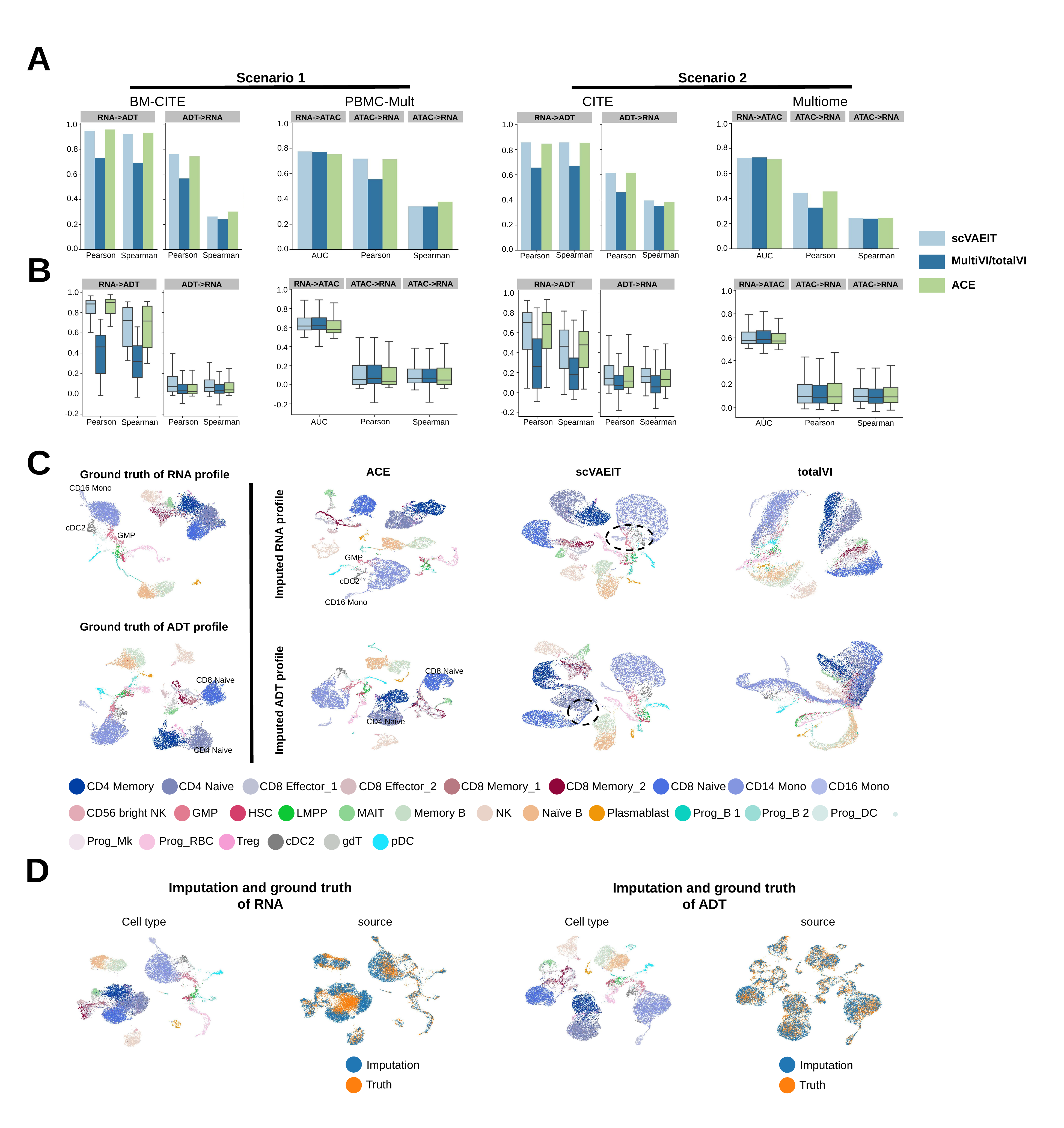

A
Scenario 2
Scenario 1
BM-CITE
PBMC-Mult
CITE
Multiome
RNA->ATAC
ATAC->RNA
ATAC->RNA
ADT->RNA
RNA->ATAC
ATAC->RNA
ATAC->RNA
RNA->ADT
ADT->RNA
RNA->ADT
1.0
1.0
1.0
1.0
0.8
0.8
0.8
0.8
0.6
0.6
0.6
0.6
0.4
0.4
0.4
0.4
0.2
0.2
0.2
0.2
scVAEIT
0.0
0.0
0.0
0.0
B
Pearson
Spearman
Spearman
Pearson
Spearman
Pearson
Spearman
Pearson
AUC
Spearman
AUC
Spearman
Pearson
Pearson
MultiVI/totalVI
ACE
ATAC->RNA
ATAC->RNA
RNA->ATAC
ADT->RNA
ADT->RNA
ATAC->RNA
ATAC->RNA
RNA->ADT
RNA->ADT
RNA->ATAC
1.0
1.0
1.0
1.0
0.8
0.8
0.8
0.8
0.6
0.6
0.6
0.6
0.4
0.4
0.4
0.4
0.2
0.2
0.2
0.2
0.0
0.0
0.0
-0.2
0.0
-0.2
-0.2
Pearson
Pearson
Pearson
AUC
Spearman
Pearson
Spearman
Pearson
Spearman
Spearman
Spearman
Pearson
AUC
Spearman
C
ACE
scVAEIT
totalVI
Ground truth of RNA profile
CD16 Mono
cDC2
GMP
Imputed RNA profile
GMP
cDC2
CD16 Mono
Ground truth of ADT profile
CD8 Naive
CD8 Naive
Imputed ADT profile
CD4 Naive
CD4 Naive
CD4 Memory
CD4 Naive
CD8 Effector_1
CD8 Effector_2
CD8 Memory_1
CD8 Memory_2
CD8 Naive
CD14 Mono
CD16 Mono
CD56 bright NK
GMP
HSC
LMPP
MAIT
Memory B
NK
Naïve B
Plasmablast
Prog_B 1
Prog_B 2
Prog_DC
Prog_Mk
Prog_RBC
Treg
cDC2
gdT
pDC
D
Imputation and ground truth of RNA
Imputation and ground truth of ADT
Cell type
source
Cell type
source
Imputation
Imputation
Truth
Truth
