# Supplementary material for: ACE: A Versatile Contrastive Learning Framework for Single-cell Mosaic Integration
Source: Genomics Proteomics Bioinformatics. 2025 Aug 4;23(4):qzaf062. doi: 10.1093/gpbjnl/qzaf062 (PMC12582371; doi:10.1093/gpbjnl/qzaf062)
Supplement: qzaf062_Supplementary_Data [file qzaf062_supplementary_data.zip › Figure S37.pptx]

## Slide 1
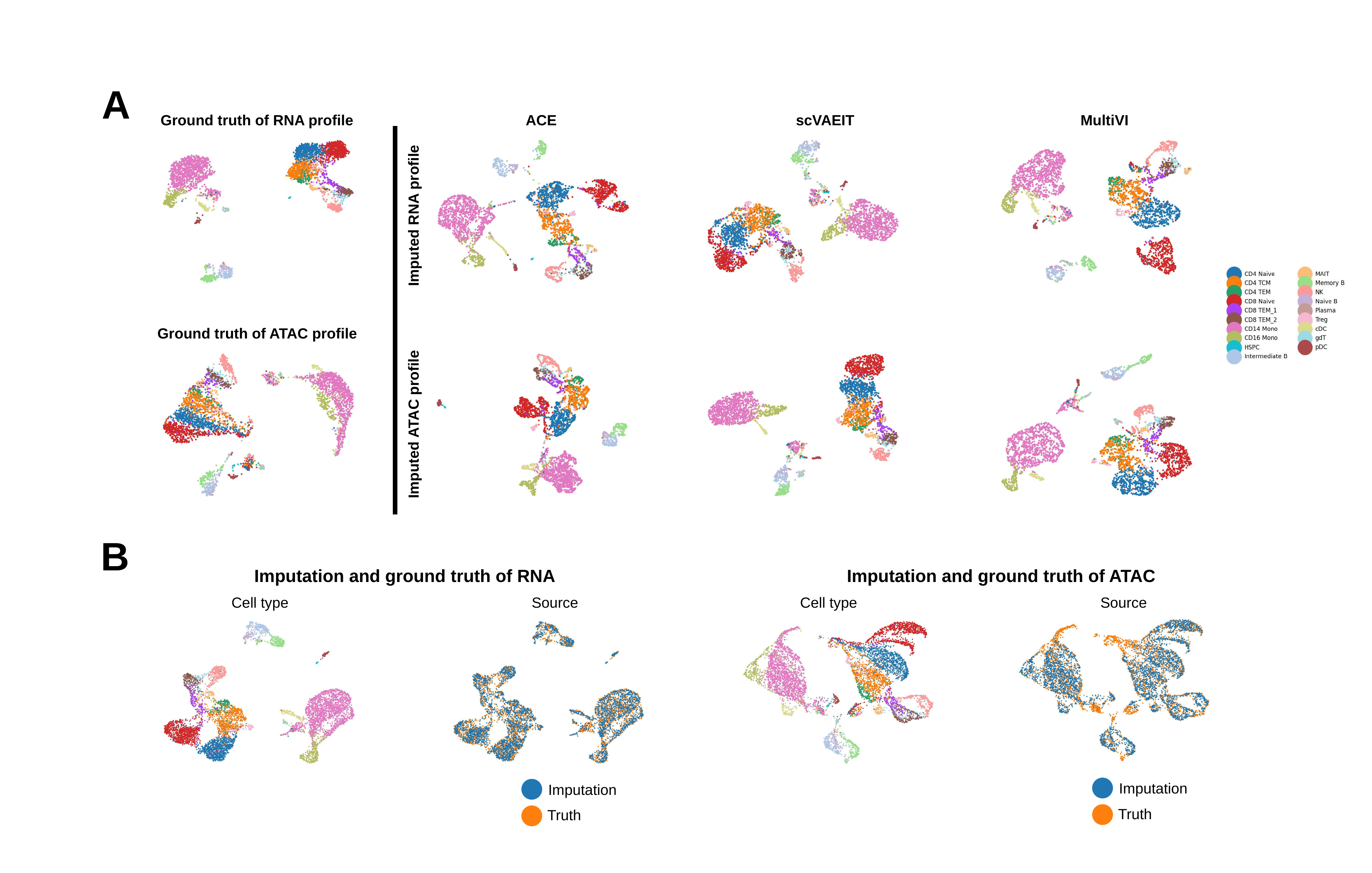

A
Ground truth of RNA profile
ACE
scVAEIT
MultiVI
Imputed RNA profile
Ground truth of ATAC profile
Imputed ATAC profile
B
Imputation and ground truth of RNA
Imputation and ground truth of ATAC
Cell type
Source
Cell type
Source
Imputation
Imputation
Truth
Truth
