# Supplementary material for: ACE: A Versatile Contrastive Learning Framework for Single-cell Mosaic Integration
Source: Genomics Proteomics Bioinformatics. 2025 Aug 4;23(4):qzaf062. doi: 10.1093/gpbjnl/qzaf062 (PMC12582371; doi:10.1093/gpbjnl/qzaf062)
Supplement: qzaf062_Supplementary_Data [file qzaf062_supplementary_data.zip › File S1.docx]

**File S1 Supplementary text for ACE**

**Supplementary note A1: details of evaluation metrics**

Biological preservation metrics compromise Normalized Mutual Information (NMI) and Adjusted Rand Index (ARI). NMI and ARI both evaluate the overlap of two clustering. Score 0 corresponds to random clustering and score 1 corresponds to the perfect match for both NMI and ARI. We performed Louvain clustering to obtain the best match between clusters and cell type labels. Louvain clustering was performed at a resolution range of 0.1 to 2 in steps of 0.1, and the clustering output with the highest NMI with the labels was used. We calculated ARI and NMI by the Python functions adjusted_rand_score() and normalized_mutual_info_score() from the scikit-learn library, respectively.

We used graph inverse Local Inverse Simpson’s Index (iLISI) as the batch correction metric. Graph iLISI score is a diversity score to assess batch mixing degree, which are computed from neighborhood lists per node from integrated k-Nearest Neighbors (kNN) graphs. Following StabMap, we not only use batch labels ($\mathrm{iLISI}_{\mathrm{batch}}$) to compute iLISI but also used modality labels ($\mathrm{iLISI}_{\mathrm{mod}}$) to compute iLISI. For example, if one batch is measured with RNA and Protein (ADT) then its modality label is RNA + ADT, whereas if one batch is measured with RNA, then its modality label is RNA. It can be regarded as evaluation at two levels of resolution, in which modality label is at coarse-grained resolution. Score 0 corresponds to separation of batches and score 1 corresponds to the perfect mixing of batches. We calculated graph iLISI scores by the Python function scib.metrics.lisi.ilisi_graph() from the scib package.

Modality alignment evaluation metrics compromise fraction of samples closer than the true match (FOSCTTM) and matching score (MS). FOSCTTM measures the alignment degree between modalities embeddings. For each modality pair ($m_{1}$, $m_{2}$), it is defined as [1]:

$$\begin{aligned} \mathrm{FOSCTT}M_{0}\left( m_{1}, m_{2} \right)=\frac{1}{2N}\left( \sum_{i} \frac{N_{i}^{m_{1}}}{N}+\sum_{i} \frac{N_{i}^{m_{2}}}{N} \right)\#\left( 1 \right) \end{aligned}$$

$$\begin{aligned} N_{i}^{m_{1}}=\left| \left\{ l \right|\left\| z_{l}^{m_{1}}-z_{i}^{m_{2}} \right\|_{2}<\left\| z_{i}^{m_{1}}-z_{i}^{m_{2}} \right\|_{2}\} \right|\#\left( 2 \right) \end{aligned}$$

$$\begin{aligned} N_{i}^{m_{2}}=\left| \left\{ l \right|\left\| z_{l}^{m_{2}}-z_{i}^{m_{1}} \right\|_{2}<\left\| z_{i}^{m_{2}}-z_{i}^{m_{1}} \right\|_{2}\} \right|\#\left( 3 \right) \end{aligned}$$

where $N$ is the number of cells that’s measured with modalities $m_{1}$ and $m_{2}$; $N_{i}^{m_{1}}$ denotes the number of cells in modality $m_{1}$ that are closer to $z_{i}^{m_{2}}$ than $z_{i}^{m_{1}}$ to $z_{i}^{m_{2}}$, and $N_{i}^{m_{2}}$ is the same; If existing three modalities in one dataset, we calculated $\mathrm{FOSCTT}M_{0}$ for every modality pair and took an average. Finally, we rescaled $\mathrm{FOSCTTM}$as 1 − $\mathrm{FOSCTT}M_{0}$, where score of 1 indicates perfect modality alignment. To calculate MS between two modalities $m_{1},m_{2}$, a cross-modality matching matrix $P$ is first constructed by computing a Jaccard index of cross-modality nearest neighbors in the aligned embedding space [1]:

$$\begin{aligned} P_{m_{1},m_{2}}\left( i,j \right)=\frac{\left| \left( NN^{m_{2}}\left( x_{i}^{m_{1}} \right)\cap NN^{m_{2}}\left( x_{j}^{m_{2}} \right) \right)\cup\left( NN^{m_{1}}\left( x_{j}^{m_{2}} \right)\cap NN^{m_{1}}\left( x_{i}^{m_{1}} \right) \right) \right|}{\left| \left( NN^{m_{2}}\left( x_{i}^{m_{1}} \right)\cup NN^{m_{2}}\left( x_{j}^{m_{2}} \right) \right)\cup\left( NN^{m_{1}}\left( x_{j}^{m_{2}} \right)\cup NN^{m_{1}}\left( x_{i}^{m_{1}} \right) \right) \right|}\#\left( 4 \right) \end{aligned}$$

where $NN^{m_{2}}(x_{i}^{m_{1}})$ denotes the set of cell $x_{i}^{m_{1}}$’s nearest neighbors in modality $m_{2}$; $NN^{m_{2}}(x_{j}^{m_{2}})$ denotes the set of cell $x_{j}^{m_{2}}$’s nearest neighbors in modality $m_{2}$; the other is the same. $|\cdot|$ denotes the number of elements in a set. Following the evaluation protocol of CLUE, the number of nearest neighbors is set to 1. The MS is computed as follows:

$$\begin{aligned} MS=\frac{1}{N}\sum_{i} \sum_{j} \tilde{P}_{m_{1},m_{2}}\left( i,j \right)\cdot\delta_{i,j}\#\left( 5 \right) \end{aligned}$$

where $\tilde{P}$ denotes row-normalized $P$. $\delta_{i,j}$ is 1 if cell $i$ and $j$ were measured in the same cell and 0 otherwise. $N$ is the number of cells. If existing three or more modalities in one dataset, we calculated MS for every modality pair and took an average. MS has a range of [0, 1], where a score of 1 indicates a perfect alignment.

**Supplementary note A2: detail of compared methods settings**

*Cobolt*

We run Cobolt following its tutorial (<https://github.com/epurdom/cobolt/blob/master/docs/tutorial.ipynb>). We first selected highly variable genes or peaks and input the count data matrix into the model for training. Most of the training hyperparameters are set to default values: training iteration = 100, latent dimension = 10, batch size = 128. The default learning rate is 0.005 but sometimes it will cause numeric issues. If so, we set the learning rate to 0.001. When executing “calc_all_latent” function on the CITE-ASAP dataset, we set the parameter “target” as “[True, True, False]”, otherwise it cannot execute normally. Since Cobolt cannot well deal with the batch effect within the same modality, we added Harmony as the post-processing step to correct its final embedding across all experiments.

*scMoMaT*

We run scMoMaT following its tutorial (<https://github.com/PeterZZQ/scMoMaT/blob/main/demo_scmomat.ipynb>). We first selected highly variable genes or peaks and input the count data matrix into the model for training. Note scMoMaT generates pseudo-count RNA matrix using scATAC-seq data, but we find this step had negative impact in most of cases. Thus, we ignored this step across all experiments. The reason is possibly that the pseudo-count RNA matrix is noisy which will introduce much noise and reduce model performance. We used the default parameter settings in all datasets except the BM-CITE dataset. For the BM-CITE dataset, we found that setting training epochs to 4000 caused overfitting so we set the training epochs to 2000.

*StabMap*

We run StabMap following its tutorial (<https://github.com/MarioniLab/StabMap/tree/main/vignettes>). We first selected highly variable genes or peaks and input the log-normalized data matrix into the method for training. All the parameters are set to default values except the “maxFeatures” parameter which we tuned it according to the total number of features across all modalities. Since StabMap cannot well deal with the batch effect within the same modality, we added Harmony as the post-processing step to correct its final embedding across all experiments.

*MatchCLOT*

We run MatchCLOT following its pipeline ([https://github.com/AI4SCR/MatchClot/tree/main/tutorials](https://github.com/AI4SCR/MatchCLOT/tree/main/tutorials)). We first trained MatchCLOT on all bi-modal datasets using following preprocessing steps: Term Frequency–Inverse Document Frequency (TF-IDF) transformation (except the protein data), log-normalization, principal component analysis (PCA) (except protein data), batch correction for each modality, respectively, using Harmony. The batch corrected low-dimensional representations or protein expression profiles are input for training. The number of max training epoch is set to 1000 and other training parameters are set to default values across all experiments. Then, we perform model inference using the same inputs as the training step and saved the final cell embeddings. Note that MatchCLOT proposes to use optimal transport (OT) method to refine the neighborhood graph among cells, which can be seen as an advanced post-processing step for model outputs (and this step can be applied to other methods as well). Considering the fairness of comparison, we did not perform this step for MatchCLOT.

*CLUE*

We run CLUE following its pipeline (<https://github.com/openproblems-bio/neurips2021_multimodal_topmethods/tree/main/src/match_modality/methods/clue>). We used the data count matrix as input and performed CLUE’s required preprocessing pipeline for different modality inputs. The preprocessing parameters followed their settings for different sequencing techniques. We first trained CLUE on the batches measured with multiple modalities. CLUE provided two groups of reference training parameters. One group is for the CITE dataset and the other one is for the Multiome dataset. The hyperparameters for training on all datasets except the Multiome dataset followed the setting on the CITE dataset. We found that even on the PBMC-Mult dataset, the setting on CITE dataset works better than the other one. After training, we saved the pretrained model weights and preprocessing parameters. Next, we performed the second round training of CLUE on the whole data of each dataset. The preprocessing parameters and training parameters followed the same settings in the first round. Since CLUE cannot well handle the batch effects within the same modality, we added Harmony as the post-processing step to correct its final embedding across all experiments.

*scVAEIT*

We run scVAEIT following its pipeline (<https://github.com/jaydu1/scVAEIT/blob/main/example.ipynb>). We first selected highly variable genes or peaks and input the log-normalized data matrix into the method for training. Following the steps for processing ATAC features, we split the peak features into multiple chunks by their chromosome ID and each chunk will be processed by a separate neural network. Note that scVAEIT can perform transductive imputation and inductive imputation. Specifically, we can train scVAEIT on the bridge batches and the batches with missing modality and perform inference on the training data. Also, we can train scVAEIT on the bridge batches first and perform inference on the batches with missing modality. We experimentally found that inductive setting led to better performance. The hyperparameters of training followed the default settings for different sequencing technologies.

*totalVI*

We run totalVI following its pipeline (<https://docs.scvi-tools.org/en/stable/tutorials/notebooks/multimodal/totalVI.html>). We first selected highly variable genes or peaks and input the count data matrix into the method for training. Similar to scVAEIT, totalVi can also perform transductive imputation and inductive imputation. We experimentally found that inductive setting led to better performance on the BM-CITE and CITE datasets. Most of training hyperparameters followed the default settings. When setting up AnnData object, we did not use “batch_key” parameter because we found that it would greatly decrease the method’s performance.

*MultiVI*

We run MultiVI following its pipeline (<https://docs.scvi-tools.org/en/stable/tutorials/notebooks/multimodal/MultiVI_tutorial.html>). We selected highly variable genes or peaks and input the log-normalized data matrix into the method for training. Similar to totalVI, we found that inductive imputation obtained better performance on the PBMC-Mult and Multiome datasets. Also, we did not use “continuous_covariate_keys” parameter because it hurt model performance.

*ACE-align and ACE-spec*

For ACE-align and ACE-spec, we developed two preprocessing pipelines tailored to different datasets.

For the raw count assays, we performed highly variable feature selection first. For the ATAC modality, we selected the top 50,000 highly variable peaks using SCANPY’s highly_variable_genes function [2], and for the RNA modality, we used the same function to select the top 10,000 highly variable genes. We then performed normalization: for ATAC peak counts, we applied a TF-IDF transformation followed by log-normalization, while for the RNA and protein modalities, we used log-normalization. Singular value decomposition (SVD) was used to decompose the normalized RNA and ATAC data into low-dimensional representations. We set the dimensionality of output as 192 for the RNA modality and 256 for the ATAC modality. The output dimensionality followed MatchCLOT’s settings [3]. Finally, we performed z-score scaling (zero-mean and standard variance) for each cell’s representation within each modality.

As in the first pipeline, we performed feature selection for the RNA and ATAC modalities using the same parameters and applied identical normalization steps for both. For the protein modality, we used centered log-ratio normalization. Finally, we used PCA to decompose the RNA and ATAC data into low-dimensional representations. The output dimensionality was set as 50.

We used “sklearn.decomposition.TruncatedSVD” from the scikit-learn package [4], an SVD implementation optimized for efficient handling of sparse matrices. We used the “arpack” SVD solver because we empirically found that it was faster. For the PCA algorithm, we used the function provided by SCANPY, scanpy.pp.pca. We used the first pipeline for theCITE, Multiome, and DOGMA datasets. The second pipeline was used for BM-CITE, PBMC-Mult, CITE-ASAP, and VPdatasets.

**Supplementary note B**

To confirm the modality gap phenomenon (*i.e.*, intra-modality embeddings of different cells can be still closer than inter-modality embeddings of the same cell after training with the Information Noise Contrastive Estimation (InfoNCE) objective), we analyzed the distance between the intra-modality embeddings and inter-modality embeddings in the BM-CITE dataset where we observed modality gap phenomenon for the InfoNCE loss. Specifically, we trained ACE-align with the InfoNCE and our proposed loss function, respectively, and used the modality-specific embeddings of the multi-modal batch (simultaneously measured with RNA and ADT) for analysis. For each cell’s RNA embedding, we calculated its distance to the paired ADT embedding (denoted as RNA–ADT), and we also picked up its nearest neighbor from the RNA embeddings (only within the multi-modal batch) and calculated the distance (denoted as RNA–RNA). We did the same thing for the cell’s ADT embedding (denoted as ADT–RNA and ADT–ADT distance).

For the embeddings derived from InfoNCE and ours, we plotted the scatter of each cell’s RNA–RNA distance and RNA–ADT distance (also for ADT–RNA and ADT–ADT distance), as shown in Figure S16. When the Manhattan distance metric was used and the temperature T was set to 0.01, we can observed in the InfoNCE’ s results that the RNA–ADT distance was much larger than RNA–RNA distance and ADT–RNA distance was much larger than ADT–ADT distance (Figure S16A), which supported our statement that intra-modality embeddings of different cells can be still closer than inter-modality embeddings of the same cell after training with the InfoNCE loss. In contrast, our proposed loss function resulted in similar embedding distance. As T increased, the distance between inter-modality embeddings derived from the InfoNCE loss became closer to that of intra-modality embeddings. This aligns with the visualization in Figure S17, which shows the modality gap shrinking as T increases. For our proposed loss, increasing T didn’t result in significant differences. When we changed the distance metric to the Euclidean distance, we still observed similar results (Figure S16B).

Moreover, we increased the neighborhood size to see if the observations remained consistent over a larger area. Specifically, for each cell’s RNA embedding, we picked up its top 10 nearest neighbors from ADT embeddings and calculated the distance (RNA–ADT distance). Also, we picked up the top 10 nearest neighbors from RNA embeddings and calculated the distance (RNA–RNA distance). We did the same thing for ADT embeddings (ADT–RNA and ADT–ADT distance). When using the Manhattan distance metric and T as 0.01, we can observe in InfoNCE’ s results that the distance between intra-modality embeddings was smaller than that of inter-modality embeddings in various neighborhood scales (Figure S18A). As for our results, the distance difference was moderate. Even if changing the distance metric and varying the temperature T, the observations remained consistent (Figure S18B).

Overall, the experimental evidence confirmed that intra-modality embeddings of different cells can be still closer than inter-modality embeddings of the same cell after training with the InfoNCE objective, while our proposed loss function can effectively reduce this modality gap.

**Supplementary note C**

To investigate the impact of the Uniform Manifold Approximation and Projection (UMAP) parameters on the visualizations, we first obtained the embeddings of ACE-align, ACE-spec, scMoMaT, and Cobolt in the BM-CITE and PBMC-Mult datasets. Then, we visualized these embeddings by varying hyperparameters of UMAP, including number of neighbors and minimum distance, following the same setting in the UMAP hyperparameter experiment [5].

The visualization results were shown in Figures S19–S22. For both datasets, we could observe that varying the hyperparameters did not affect the observed batch mixing quality. Specifically, ACE-align and ACE-spec well mixed three batches across all parameter settings whereas scMoMaT only mixed batches 1 and 3 for both datasets (Figures S19 and S21). For the PBMC-Mult dataset, Cobolt mixed batches 1 and 3 while a portion of batch 2 was isolated (Figure S21). This pattern was consistent across all parameter settings. However, the observed heterogeneity (cell types) was slightly affected by the UMAP parameters. Specifically, within each method’s visualizations, increasing the number of neighbors would make each cluster of cell type look smaller (Figures S20 and S22). Consequently, those separated cell types look more isolated due to the increased inter-cluster distance. The partially overlapping cell types became increasingly difficult to distinguish due to the decreased scale of separation boundaries (similar to zooming out). Conversely, increasing the minimum distance would make each cluster of cell type look larger. The distance between each pair of cell types decreased, while the partially overlapping cell types became more distinguishable due to the increasing scale of separation boundaries (similar to zooming in). Therefore, adjusting the hyperparameters of UMAP is similar to viewing cellular heterogeneity from different scales. However, the embedding outputs from integration methods basically determined the cellular heterogeneity that we can observe. For example, in the BM-CITE and PBMC-Mult datasets, naïve B cells and memory B cells are two slightly overlapping clusters in ACE-spec’s visualization (min_dist = 0.0125, n_neighbors = 5) (Figures S20 and S22). We could still distinguish between these two cell types after adjusting the hyperparameters. However, these two cell types were mixed in scMoMaT’s visualization and varying the parameters did not enable separation between them (Figures S20 and S22). In the PBMC-Mult dataset, CD4 Central Memory T cells (TCM) and CD4 Effector Memory T cells (TEM) cells were separable in ACE-spec’s visualizations while they were mixed in scMoMaT’s visualizations (Figure S22). In addition, we observed that the small number of neighbors resulted in many isolated small clusters, which is a common phenomenon for ACE-align/ACE-spec, scMoMaT, and Cobolt (Figures S20 and S22). This is unsurprising because small numbers of neighbors make model focus on small neighborhoods and the visualization become more dispersed.

Furthermore, we explored the hyperparameters of UMAP for visualizing ACE-spec’s embeddings on the CITE-ASAP and VP datasets. We observed that the visualizations with different hyperparameters consistently aligned with the original cell type annotations and the clustering labels (Figures S23 and S24). For example, clusters 11, 14, 15, and 16 in the CITE-ASAP dataset were rare clusters but were clearly separable across the parameter settings (Figure S23B). Clusters 14, 16, 17, and 20 were rare clusters in the VP-RNA dataset but were also separable across the parameter settings (Figure S24B). These results again demonstrated the robustness of the observed cellular heterogeneity.

We concluded that the embedding outputs from integration methods primarily determined the cellular heterogeneity that we can observe. UMAP visualization is an approach to represent the heterogeneity in a 2-dimensional space. Adjusting the hyperparameters of UMAP is analogous to zooming in or zooming out on the data and our hyperparameter analysis illustrated the stability and robustness of the observed cellular heterogeneity.

**Supplementary note D**

Ablation study of our proposed contrastive learning objective is below.

To investigate the effectiveness of our proposed learning objective for ACE-align, we compared it with InfoNCE loss on BM-CITE and CITE datasets. First, by keeping other parameters the same, we evaluated the two loss functions for different settings of temperature values. Figure S25A shows that on BM-CITE dataset, our proposed loss function clearly outperforms InfoNCE for different temperature values with respect to bio-conservation and batch correction metrics. Especially when the temperature is small, the improvement of our loss function is more significant. As for the modality alignment metrics, FOSCTTM and MS, two loss functions have similar performance. This is attributable to the fact that these two metrics solely quantify inter-modality relationships, neglecting intra-modality relationships. Consequently, our proposed loss function is not expected to exhibit enhanced performance. On the CITE dataset, the improvement of our loss function is also clear with respect to batch correction metrics and bio-conservation metrics (Figure S25B). UMAP plots on two datasets also demonstrate that InfoNCE results in modality gap phenomena when T is smaller than 0.02, whereas our proposed loss function consistently mixes all batches and modalities (Figure S17). For the reason why the bridge batch mixed well with RNA-modal batch, this is because we only use RNA modality of bridge batch to infer its final embeddings.

However, we also noticed that the improvement of our loss function on CITE dataset is lower than on the BM-CITE dataset. We hypothesized that this is because the protein modality in CITE dataset contains more features (n = 134) than in BM-CITE dataset (n = 25). More protein features indicate that the information content between two modalities is more balanced. Thus, it’s easier to avoid modality gap phenomenon on CITE dataset. To investigate how two loss functions perform with different number of protein features, we randomly selected 10, 20, 40, and 80 protein features from CITE dataset and evaluated two loss functions in different cases. Each selection was repeated three times to avoid randomness. We set the T = 0.1 for both losses to maximize the performance of InfoNCE. Figure S25C shows that for different numbers of protein features, our proposed loss function still outperforms InfoNCE with a clear margin of batch correction metrics. This indicates that our proposed loss function can better handle the cases with greatly imbalanced information content between modalities, thereby enhancing its generalization capabilities.

**Supplementary note E**

Removing batch effects prior to model training has a notable impact on the model’s performance [6]. We selected Harmony [7] as the batch correction method because it has been validated as a robust solution in numerous studies. To assess the impact of different batch correction methods on the model’s performance, we compared three commonly used batch correction methods, mutual nearest neighbors (MNN) [8], Scanorama [9], Harmony [7], and a new method, PARE [10] (as suggested by the reviewer), on the CITE, Multiome, and DOGMA datasets, all of which contain batch effects within individual modality. We found that PARE required substantial computational resources, resulting in memory overflow on the CITE and Multiome datasets. To incorporate PARE for comparison, we subsampled cells from the CITE and Multiome datasets with sampling ratios of 5% and 10% (we named these datasets CITE-5%, CITE-10%, Multiome-5%, and Multiome-10%). On the full CITE and Multiome datasets, we compared MNN, Scanorama, and Harmony. On the DOGMA, CITE-5%, CITE-10%, Multiome-5%, and Multiome-10% datasets, we compared all four batch correction methods. Results were shown in Figures S26–S31.

For the four subsampled datasets, ACE-spec combined with each of the four batch correction methods yielded similar NMI scores (ACE-align showing a similar trend), while the impact on ARI scores was more notable (Figure S26). For ACE-align, Scanorama led to more robust and higher ARI scores, followed by MNN, while PARE and Harmony had similar ARI scores. For ACE-spec, Scanorama and Harmony led to higher ARI scores. PARE led to the lowest ARI scores. In terms of batch correction metrics (modal iLISI and batch iLISI), Harmony led to the highest scores for both ACE-align and ACE-spec across the four subsampled datasets, except for the Multiome-10% dataset, where ACE-spec with PARE attained the highest modal iLISI score (Figure S26). ACE-spec with PARE consistently ranked in the top two for both modal iLISI and batch iLISI scores across all four datasets. For ACE-align, MNN, Scanorama, and PARE had similar iLISI scores. In terms of modality alignment metrics (FOSCTTM and MS), four batch correction methods’ scores were close (Figure S26). To provide a more intuitive understanding of the effects of the four batch correction methods, we plotted their resulting UMAPs for the CITE-5% and Multiome-5% datasets, as shown in Figure S27. Harmony’s output displayed a more uniform mixing of batches within each modality, with well separated cell types, which was consistent with the comparison results. The other three methods had similar visualization results, where batches were mixed and the cellular heterogeneity was preserved.

For the DOGMA dataset, Harmony attained the highest NMI and ARI for ACE-align and ACE-spec among the four batch correction methods, and outperformed other integration methods (Figure S28A). PARE achieved the highest modal iLISI and batch iLISI scores for ACE-spec and followed by Harmony, which were tied with the highest scores of other integration methods. ACE-align achieved the highest iLISI scores with Harmony. The four batch correction methods led to similar FOSCTTM scores for ACE-align and ACE-spec, and all surpassed other integration methods (Figure S28A). However, Harmony attained the highest MS for ACE-align and ACE-spec, and significantly outperformed other integration methods. We visualized the batch corrected outputs from four batch correction methods using UMAP. Harmony mixed batches better within each modality, which helped to guide the modality alignment across batches (Figure S29). In terms of the overall scores, combining the four batch correction methods with ACE-spec all outperformed other state-of-the-art integration methods (Figure S28B). For ACE-align, Harmony, MNN, and PARE also achieved competitive overall performance. For the CITE dataset, ACE-spec with MNN, Scanorama, and Harmony achieved the highest overall scores among all integration methods (Figure S30). For the Multiome dataset, Harmony helped ACE-align and ACE-spec achieve the highest overall scores while MNN and Scanorama also achieved competitive performance (Figure S31).

Together, we conducted an exploration for the impact of four batch correction methods on our proposed framework. We concluded that using different batch correction methods did have an impact on the performance, and the performance differences were mainly reflected in the batch correction and modality alignment. Generally, Harmony had the best batch correction performance and consequently resulted in better modality alignment across batches. PARE achieved close or even higher batch correction scores as Harmony but still showed batch separation in the UMAP visualizations. Additionally, one limitation of PARE was that it took much longer to finish the integration than the other three methods. For instance, Harmony finished integration for DOGMA dataset within 2 min whereas PARE took 48 h to finish on our server. However, from the perspective of overall scores, all four batch correction methods helped ACE-align and ACE-spec achieve top performance among all integration methods, which demonstrated the robustness and generalizability of our proposed framework.

**Supplementary note F**

To assess whether different clustering algorithms may introduce biases in performance evaluation, we tested two additional clustering algorithms: Leiden and K-means. Leiden is an improved shared nearest neighbor clustering (SNN)-based algorithm that addresses certain limitations of the Louvain algorithm, such as disconnected communities and instability in the results. K-means, a widely used clustering method, is known for its computational efficiency and effectiveness with spherical, well-separated clusters, but it is less suited for data with irregular shapes or overlapping clusters. On the BM-CITE, PBMC-Mult, CITE, and Multiome datasets, we applied three clustering algorithms to the embeddings generated by each integration method and assessed the clustering performance using NMI and ARI. We also calculated two unsupervised metrics for each method’s embeddings, silhouette coefficient and Davies-Bouldin index (DBI), which considers both within-cluster and between-cluster distance. Following the single-cell Integration Benchmarking (scIB) benchmarking [11], the unsupervised metrics were calculated using cell type labels. Silhouette coefficient has a value range from −1 to 1, where −1 indicates strong misclassification, and +1 indicates dense and well-separated clusters. DBI has a value from 0 to infinity, and values closer to zero indicate a better cluster separation.

Using the Louvain and Leiden clustering algorithms, ACE-spec achieved the highest NMI and ARI scores on the BM-CITE, CITE, and Multiome datasets (Figure S32). Using Leiden algorithm, ACE-align ranked in the top three for NMI and ARI scores on BM-CITE, PBMC-Mult, and Multiome datasets. Cobolt and CLUE using the Louvain and Leiden algorithms showed NMI scores comparable to ACE-spec while StabMap showed the lowest NMI and ARI scores among all methods. When using the K-means clustering algorithm, the NMI and ARI scores of ACE-align and ACE-spec both dropped compared to using Louvain and Leiden (Figure S32). This is unsurprising because the UMAP visualizations suggested that many cell types in ACE-align’s and ACE-spec’s embedding space exhibited non-convex shapes (Figures S4 and S8), which are challenging for the K-means algorithm to accurately capture. Nevertheless, ACE-spec still achieved competitive NMI and ARI scores among all methods while ACE-align’s ARI scores were relatively lower. For other integration methods, we also observed performance drop when changing the clustering algorithm as K-means, especially for ARI scores (Figure S32). However, CLUE and CLUE-Harmony achieved higher NMI and ARI scores with K-means on PBMC-Mult dataset. To compare the best clustering performance of each integration method, we selected the clustering algorithm that achieved the highest NMI score on each dataset and compared all methods’ performance again. The results showed that ACE-spec achieved the highest NMI and ARI scores on the BM-CITE, CITE, and Multiome datasets, while ACE-align ranked among the top three for NMI and ARI on the BM-CITE, PBMC-Mult, and Multiome datasets (Figure S33).

In terms of silhouette scores, ACE-align ranked second on the BM-CITE dataset, behind Cobolt (Figure S34A). On the other datasets, CLUE achieved the highest silhouette scores, followed by Cobolt. Across all four datasets, ACE-spec’s silhouette scores were lower than those of the top-performing methods. Regarding the DBI (lower score indicates better embedding quality), ACE-align achieved the lowest DBI on the BM-CITE dataset, followed by Cobolt and CLUE (Figure S34B). On the other datasets, ACE-align, Cobolt, and CLUE consistently ranked in the top three. However, ACE-spec exhibited higher DBI scores compared to the leading methods. The poor unsupervised metric scores of ACE-spec, particularly for the CITE and Multiome datasets, can be attributed to the distortion of many clusters in ACE-spec’s embedding space. Based on the UMAP visualizations, these clusters appeared “stretched and squeezed” (Figure S8), which increased within-cluster distance and decreased the between-cluster distance. However, this deformation may reflect genuine developmental trajectories. For example, in both the CITE and Multiome datasets, there existed an erythrocyte development trajectory involving hematopoietic stem cell (HSC), megakaryocyte and erythrocyte (MK/E) progenitor, proerythroblast, erythroblast, normoblast, and reticulocyte [12]. In ACE-spec’s embeddings, these cell types formed a continuous and stretched structure, consistent with the pseudotime that was annotated in the original study [12] (Figure S35). In contrast, Cobolt and other methods presented this trajectory less clearly. Therefore, although the stretched clusters resulted in lower unsupervised metric scores for ACE-spec, this pattern accurately captured the underlying biological trajectories.

Overall, ACE-align and ACE-spec achieved superior NMI and ARI scores with SNN-based clustering algorithms but showed decreased scores with the K-means algorithm. ACE-align outperformed ACE-spec in terms of unsupervised metric scores, but both were relatively poorer than the best-performing methods.

**Supplementary note G**

Evaluation of ACE’s performance in imputing missing molecular layers is below.

The goals of mosaic integration are not only focused on projecting different batches into a consensus space, but also help impute raw features for those unmeasured modalities in batches as a natural biproduct of integration procedure [13,14]. We realized that our cross-modal matching strategy can be applied to impute the missing molecular layers, as described in the Materials and methods section. To evaluate its performance, we organized two scenarios. In both scenarios, there exist bridge batches and multiple batches measured with the same one modality. In scenario 1, batch effects do not exist between bridge batches and single-modal batches. Accordingly, BM-CITE dataset and PBMC-Multiome dataset are used for scenario 1, and the methods are required to reconstruct the missing profiles from the available profiles, which includes four subtasks: predicting RNA from protein (ADT$->$RNA), predicting protein from RNA (RNA$->$ADT), and predicting RNA from ATAC (ATAC$->$RNA), and predicting ATAC from RNA (RNA$->$ATAC). In scenario 2, batch effects exist between bridge batches and single-modal batches. CITE dataset and Multiome dataset are used in this case, where the same four prediction subtasks are included. Following scVAEIT, we used Spearman correlation coefficient (SCC) and Pearson correlation coefficient (PCC) as evaluation metrics of RNA and protein imputation, and used area under the receiver operating characteristic curve (AUROC) as the evaluation metric of ATAC imputation. The overall score of each metric is computed by concatenating all cell’s features into one vector. The totalVI, MultiVI, and scVAEIT were evaluated for comparisons. The totalVI and MultiVI are restricted to bimodal analysis, in which totalVI jointly models RNA and proteins and MultiVI jointly models RNA and ATAC. Detailed method settings can be found in Supplementary note A2.

In scenario 1, ACE and scVAEIT show similar overall scores of different metrics across four subtasks, and they outperform totalVI/MultiVI with a distinct margin in subtasks of RNA$->$ADT, ADT$->$RNA, and ATAC$->$RNA (Figure S36A). Evaluation on each feature separately also shows that ACE and scVAEIT achieve similar metric scores and they are more robust than toTalVI/MultiVI (Figure S36B). Next, we visualized cells with their imputed features using UMAP. Figure S36C shows that ACE’s imputed RNA and protein profiles on BM-CITE dataset well preserve the original cellular heterogeneity. When visualizing the imputed features and the ground truth of missing modalities together, it’s observed that they overlap well (Figure S36D). Compared to the imputed features of scVAEIT, we found that ACE’s imputations show better separation of cell types (Figure S36C). For instance, within ACE’s imputed RNA profiles, CD16 mono cells are distinct from Granulocyte-Monocyte Progenitor cells (GMP) and Conventional Dendritic Cell type 2 (cDC2) cells, whereas in scVAEIT’s imputations, CD16 mono cells overlapped with these two cell types. Within the ground truth of missing RNA profiles, these three types are also separated. CD4 naïve and CD8 naïve are two separated clusters within ACE’s imputed protein profiles while they are overlapped within scVAEIT’s imputations. The ground truth of protein profiles also shows that these two types are separated. UMAP plots on PBMC-Multiome dataset also demonstrate ACE’s capability in reconstructing raw omics features (Figure S37). Nevertheless, these visualizations also reveal that ACE’s imputation is not smoothing enough compared to the ground truth, which is an inherent limitation of ACE due to discrete imputation process.

In scenario 2, batch effects exist between bridge batches and single-modal batches. Although we can correct the batch effects within the shared modality, it’s highly possible that the imputations of missing modality still have batch effects with the ground truth. Evaluation results are shown in Figure S36A and B. ACE shows similar performance to scVAEIT and they generally outperform totalVI/MultiVI. However, compared to scenario 1, the metric scores dropped clearly. For example, ACE’s PCC of protein imputation on CITE dataset dropped by 11% compared to the BM-CITE dataset (PCC dropped by 17% for the RNA imputation). On the same dataset, scVAEIT’s PCC of protein imputation dropped by 9% and PCC of RNA imputation dropped by 19%, indicating that batch effects have a notable impact on the quality of imputation. We visualized cells with the imputed features and cells with the ground truth of missing profiles together and observed that those cells with imputed features are separated to those cells using real features (Figures S38 and S39). The obvious separation can also be observed in scVAEIT’s results. We think that this is a common problem for existing reconstruction methods and leave this problem for future work.

**Reference:**

[1] Tu X, Cao ZJ, Xia C, Mostafavi S, Gao G. Cross-linked unified embedding for cross-modality representation learning. Proc 36th Int Conf NeuralIPS 2022:15942–55.

[2] Wolf FA, Angerer P, Theis FJ. SCANPY: large-scale single-cell gene expression data analysis. Genome Biol 2018;19:15.

[3] Gossi F, Pati P, Chouvardas P, Martinelli AL, Kruithof-de Julio M, Rapsomaniki MA. Matching single cells across modalities with contrastive learning and optimal transport. Brief Bioinform 2023;24:bbad130.

[4] Pedregosa F, Varoquaux G, Gramfort A, Michel V, Thirion B, Grisel O, et al. scikit-learn: machine learning in Python. J Mach Learn Res 2011;12:2825–30.

[5] McInnes L, Healy J, Melville J. UMAP: uniform manifold approximation and projection for dimension reduction. arXiv 2018; https://doi.org/10.48550/arXiv.1802.03426.

[6] Hicks SC, Townes FW, Teng M, Irizarry RA. Missing data and technical variability in single-cell RNA-sequencing experiments. Biostatistics 2018;19:562–78.

[7] Korsunsky I, Millard N, Fan J, Slowikowski K, Zhang F, Wei K, et al. Fast, sensitive and accurate integration of single-cell data with Harmony. Nat Methods 2019;16:1289–96.

[8] Haghverdi L, Lun ATL, Morgan MD, Marioni JC. Batch effects in single-cell RNA-sequencing data are corrected by matching mutual nearest neighbors. Nat Biotechnol 2018;36:421–7.

[9] Hie B, Bryson B, Berger B. Efficient integration of heterogeneous single-cell transcriptomes using Scanorama. Nat Biotechnol 2019;37:685–91.

[10] Chen AA, Clark K, Dewey BE, DuVal A, Pellegrini N, Nair G, et al. PARE: a framework for removal of confounding effects from any distance-based dimension reduction method. PLoS Comput Biol 2024;20:e1012241.

[11] Luecken MD, Buttner M, Chaichoompu K, Danese A, Interlandi M, Mueller MF, et al. Benchmarking atlas-level data integration in single-cell genomics. Nat Methods 2022;19:41–50.

[12] Luecken MD, Burkhardt DB, Cannoodt R, Lance C, Agrawal A, Aliee H, et al. A sandbox for prediction and integration of DNA, RNA, and proteins in single cells. 35th Conf NeuralIPS Datasets and Benchmarks Track (Round 2) 2021.

[13] Argelaguet R, Cuomo ASE, Stegle O, Marioni JC. Computational principles and challenges in single-cell data integration. Nat Biotechnol 2021;39:1202–15.

[14] Du JH, Cai Z, Roeder K. Robust probabilistic modeling for single-cell multimodal mosaic integration and imputation via scVAEIT. Proc Natl Acad Sci U S A 2022;119:e2214414119.
