# Supplementary figures and images for: ACE: A Versatile Contrastive Learning Framework for Single-cell Mosaic Integration
Source: Genomics Proteomics Bioinformatics. 2025 Aug 4;23(4):qzaf062. doi: 10.1093/gpbjnl/qzaf062 (PMC12582371; doi:10.1093/gpbjnl/qzaf062)

## Slide 1
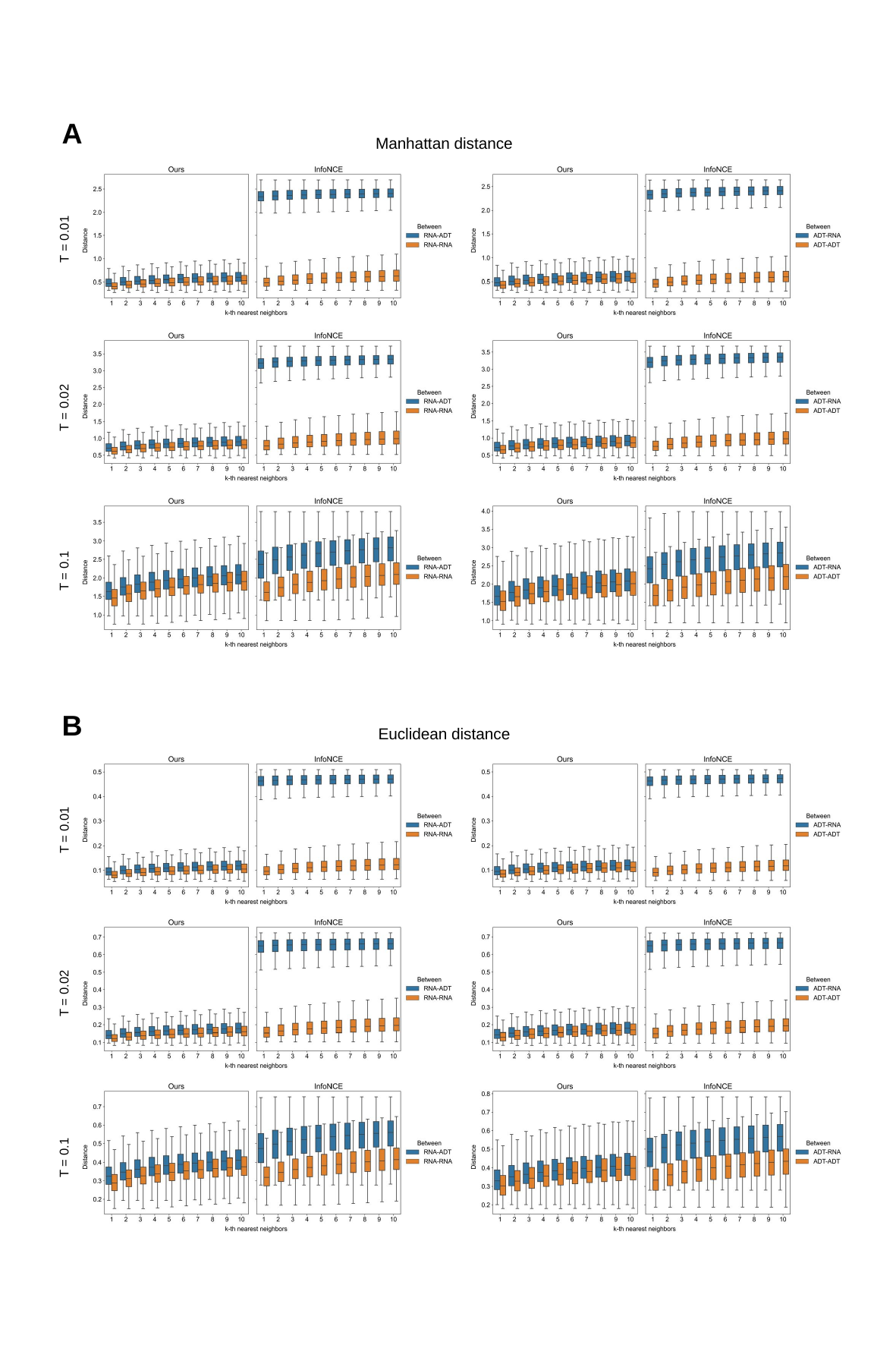

A
Manhattan distance
T = 0.01
T = 0.02
T = 0.1
B
Euclidean distance
T = 0.01
T = 0.02
T = 0.1

Supplement: qzaf062_Supplementary_Data [file qzaf062_supplementary_data.zip › Figure S18.pptx]

## Slide 1
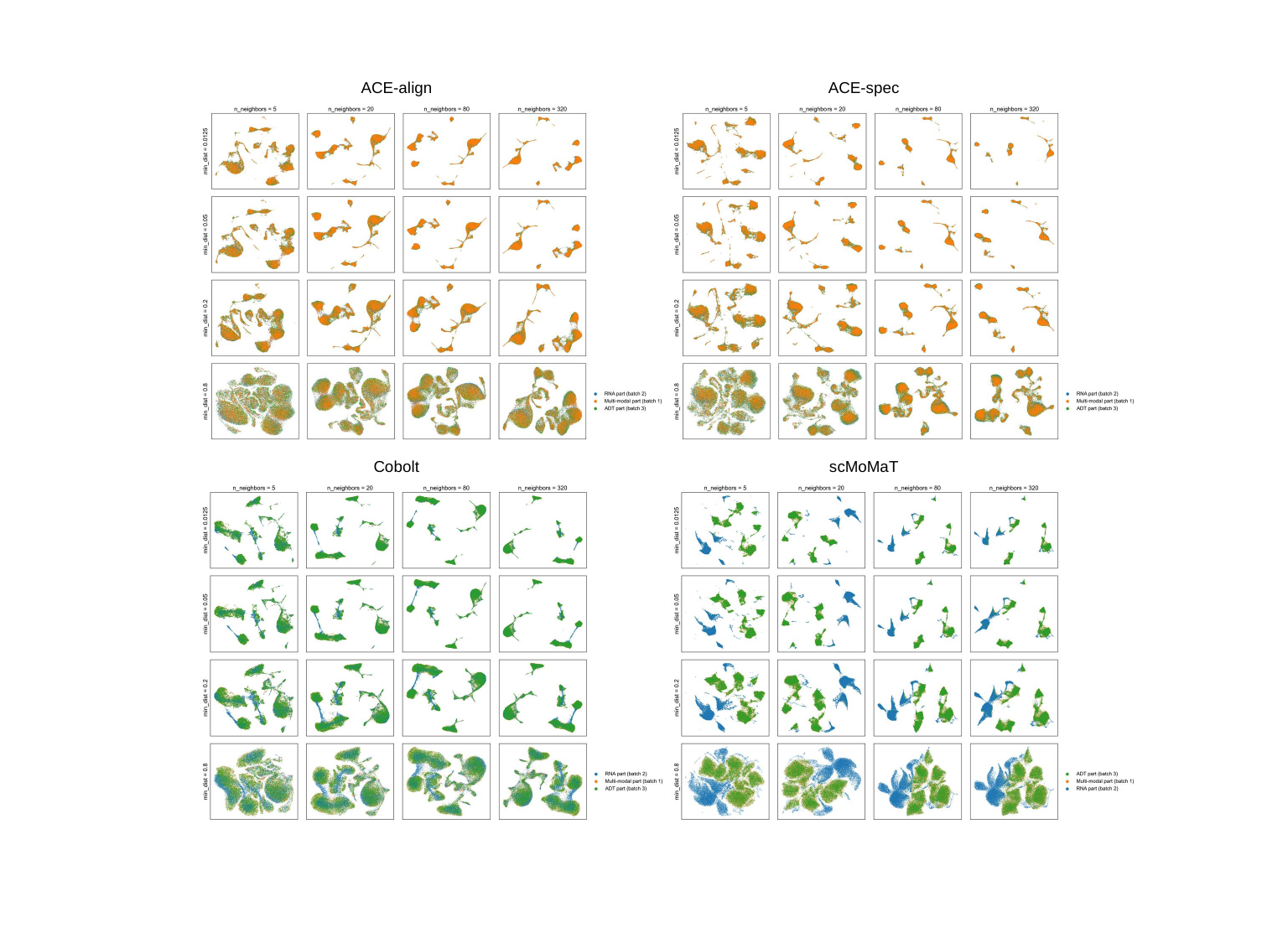

ACE-align
ACE-spec
Cobolt
scMoMaT

Supplement: qzaf062_Supplementary_Data [file qzaf062_supplementary_data.zip › Figure S19.pptx]

## Slide 1
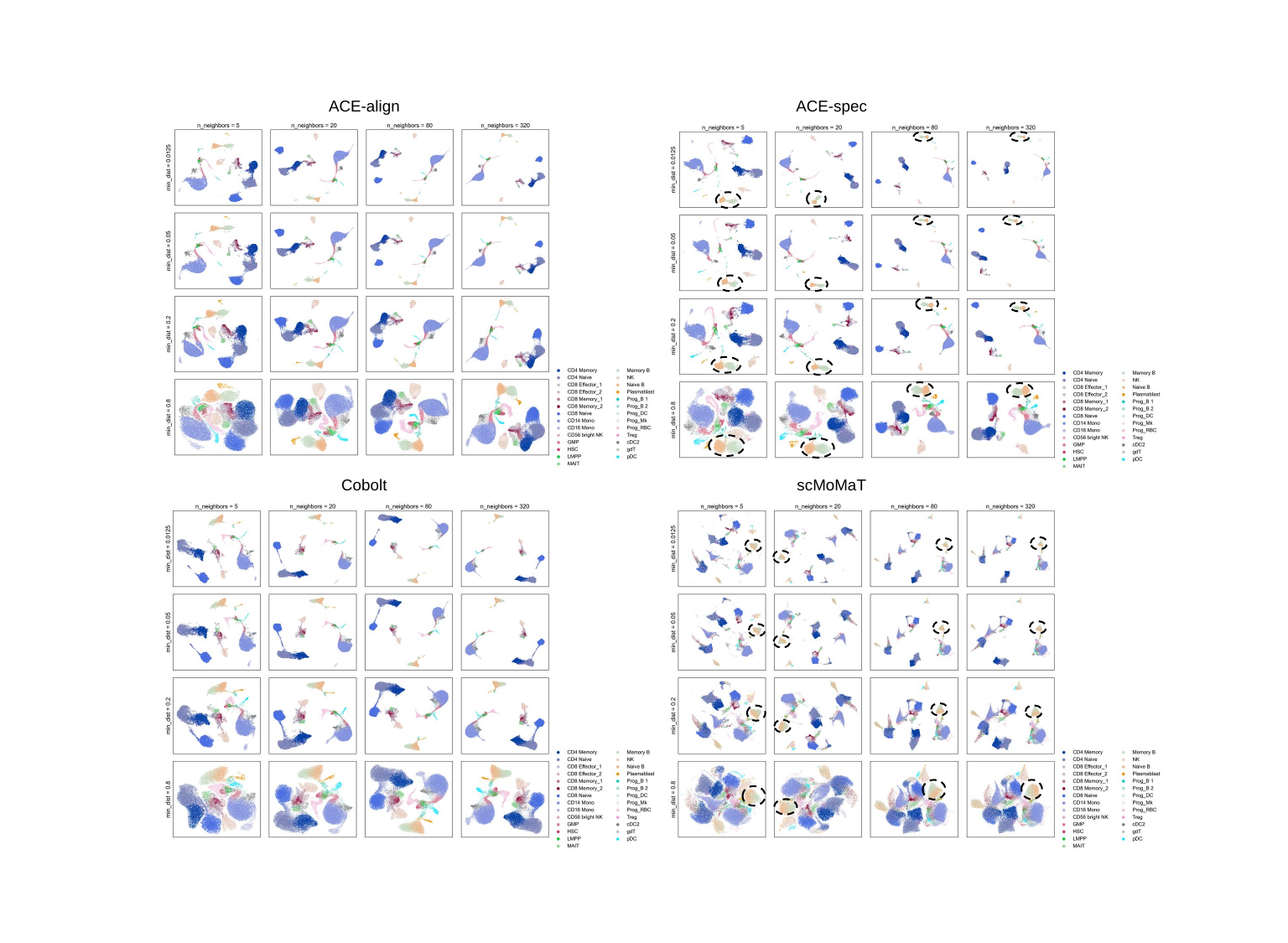

ACE-align
ACE-spec
Cobolt
scMoMaT

Supplement: qzaf062_Supplementary_Data [file qzaf062_supplementary_data.zip › Figure S20.pptx]

## Slide 1
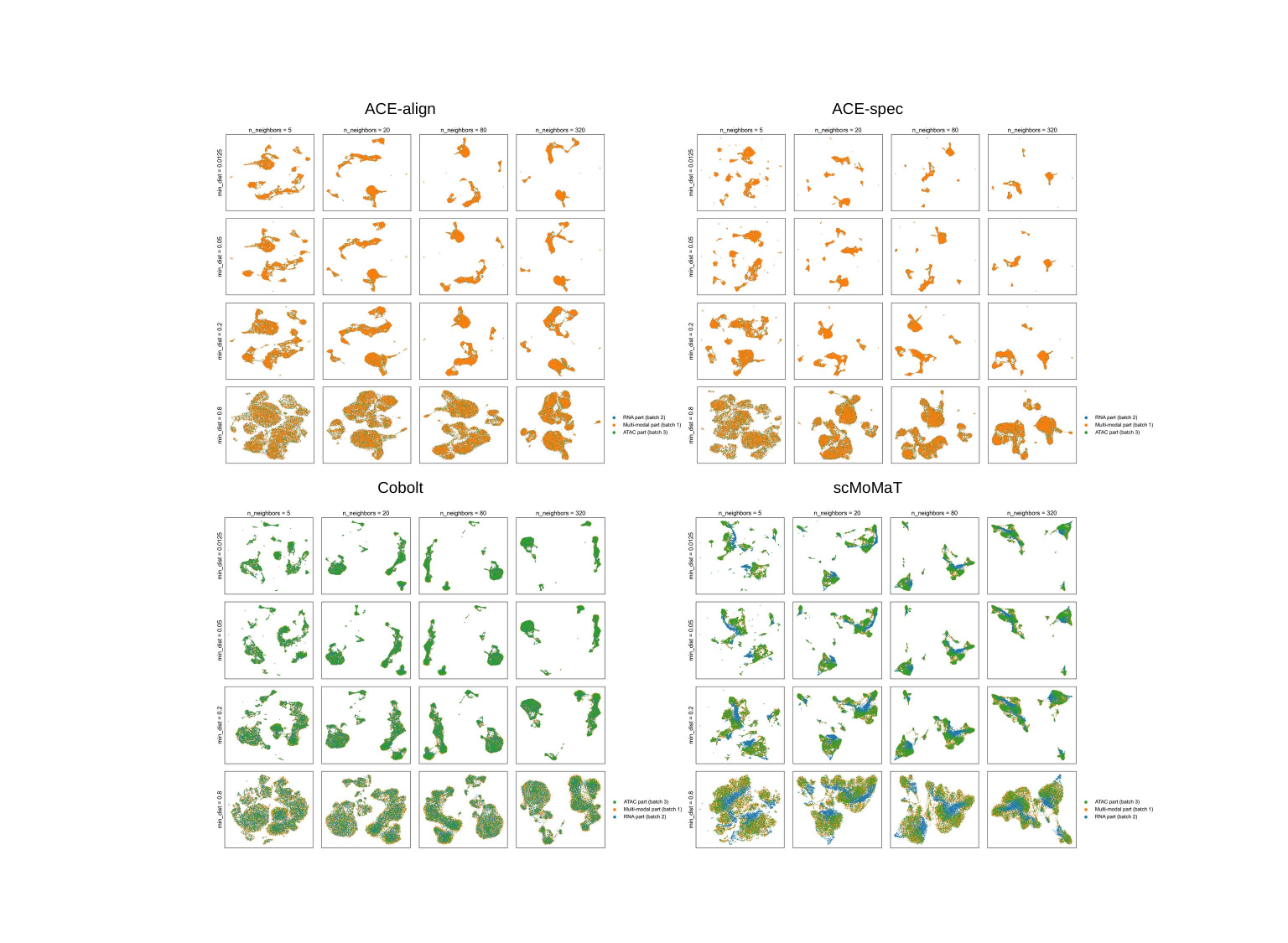

ACE-align
ACE-spec
Cobolt
scMoMaT

Supplement: qzaf062_Supplementary_Data [file qzaf062_supplementary_data.zip › Figure S21.pptx]

## Slide 1
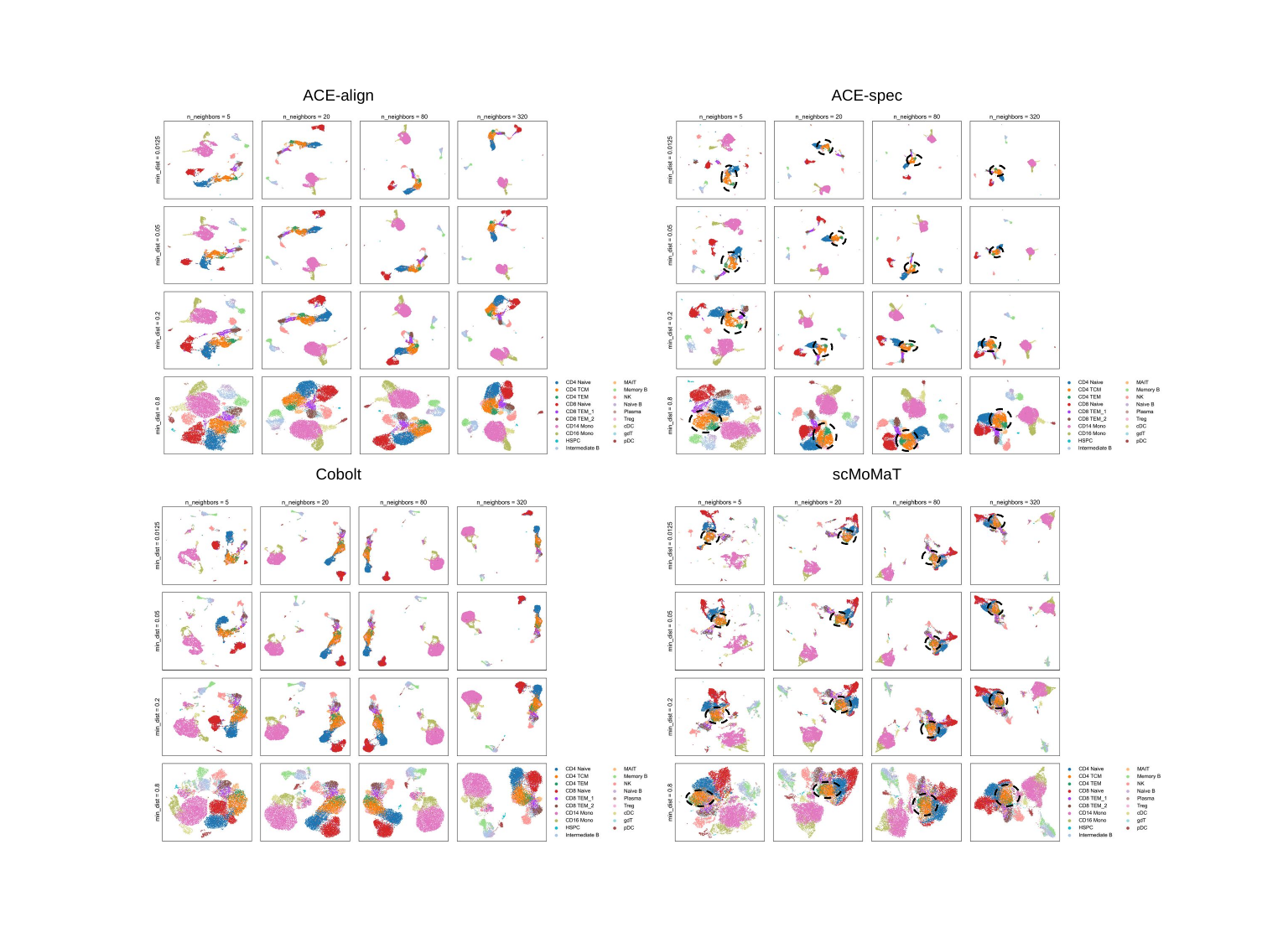

ACE-align
ACE-spec
Cobolt
scMoMaT

Supplement: qzaf062_Supplementary_Data [file qzaf062_supplementary_data.zip › Figure S22.pptx]

## Slide 1
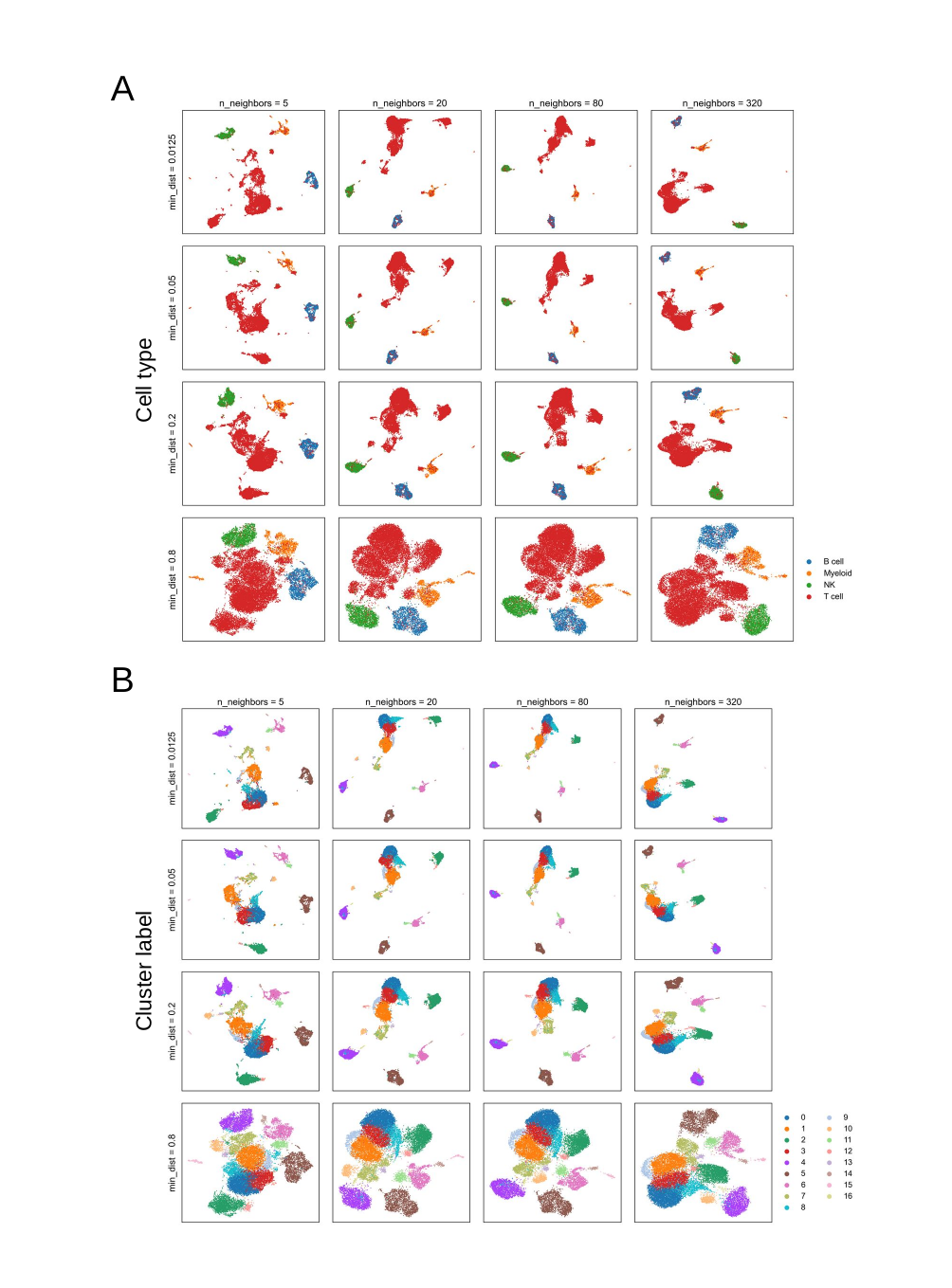

A
Cell type
Cluster label
B

Supplement: qzaf062_Supplementary_Data [file qzaf062_supplementary_data.zip › Figure S23.pptx]

## Slide 1
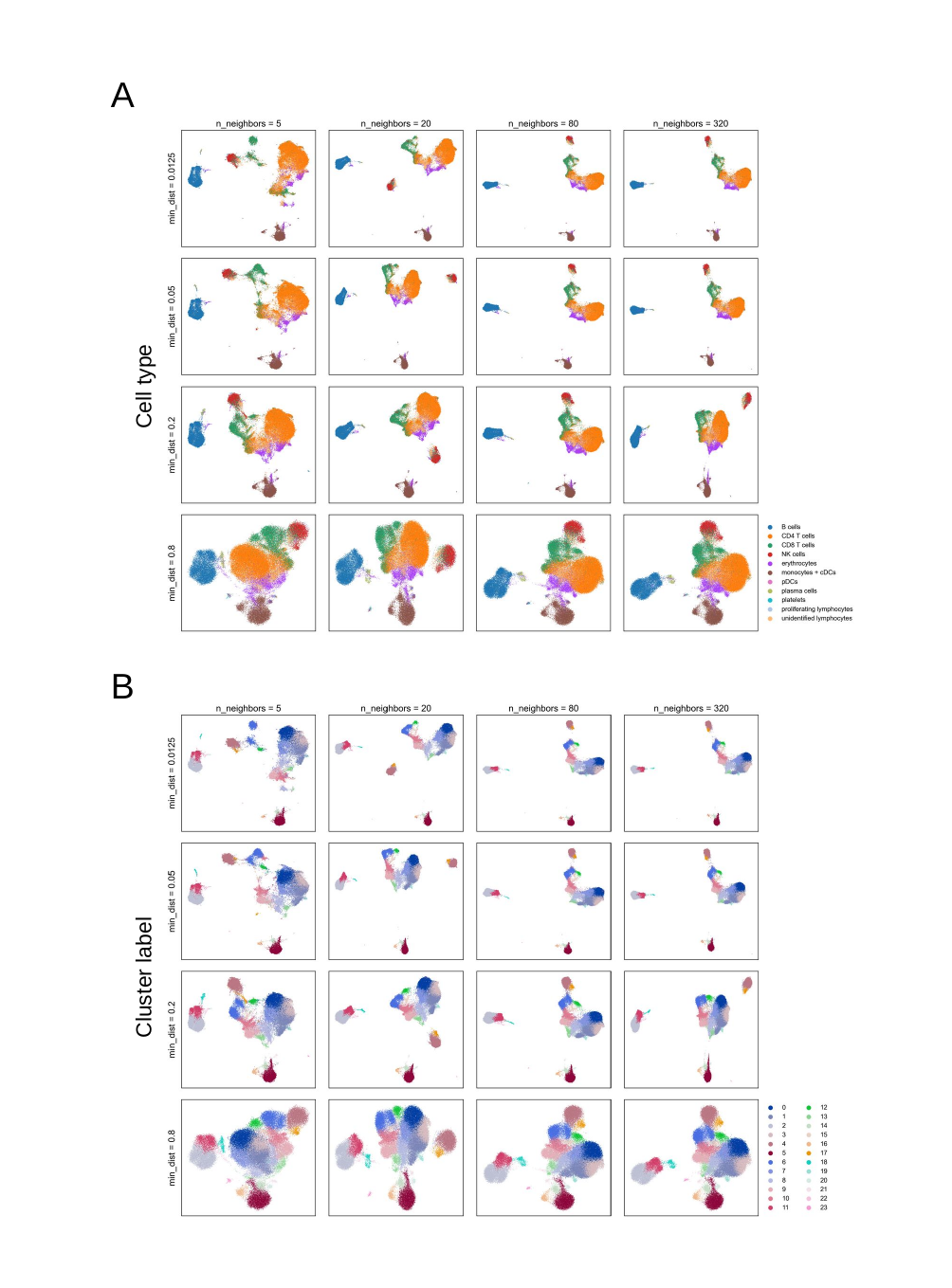

A
Cell type
B
Cluster label

Supplement: qzaf062_Supplementary_Data [file qzaf062_supplementary_data.zip › Figure S24.pptx]

## Slide 1
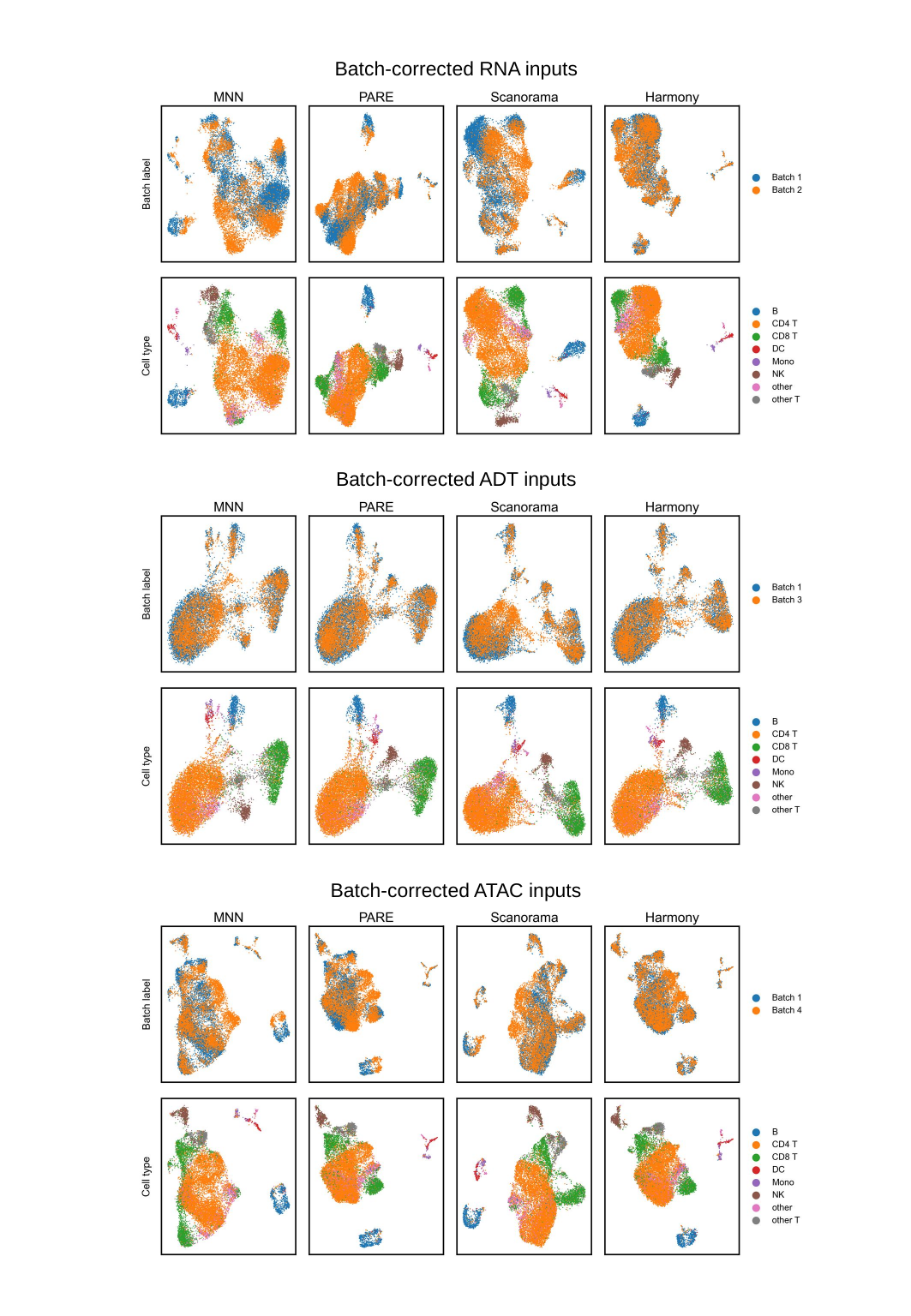

Batch-corrected RNA inputs
Batch-corrected ADT inputs
Batch-corrected ATAC inputs

Supplement: qzaf062_Supplementary_Data [file qzaf062_supplementary_data.zip › Figure S29.pptx]
